# A THEMIS:SHP1 complex promotes T-cell survival

**DOI:** 10.15252/embj.201387725

**Published:** 2014-12-22

**Authors:** Wolfgang Paster, Annika M Bruger, Kristin Katsch, Claude Grégoire, Romain Roncagalli, Guo Fu, Nicholas RJ Gascoigne, Konstantina Nika, Andre Cohnen, Stephan M Feller, Philip C Simister, Kelly C Molder, Shaun-Paul Cordoba, Omer Dushek, Bernard Malissen, Oreste Acuto

**Affiliations:** 1 T Cell Signalling Laboratory, Sir William Dunn School of Pathology, University of OxfordOxford, UK; 2 Centre d'Immunologie de Marseille-Luminy, UM2 Aix-Marseille UniversitéMarseille, France; 3 INSERM U1104Marseille, France; 4 CNRS UMR7280Marseille, France; 5 Department of Immunology and Microbial Science, The Scripps Research InstituteLa Jolla, CA, USA; 6 Department of Microbiology, Yong Loo Lin School of Medicine, National University of SingaporeSingapore, Singapore; 7 Biological Systems Architecture Group, Department of Oncology, Weatherall Institute of Molecular Medicine, University of OxfordOxford, UK; 8 Tumor Biology Unit, Institute of Molecular Medicine, ZAMED, Martin Luther University Halle-WittenbergHalle, Germany; 9 Molecular Immunology Group, Sir William Dunn School of Pathology, University of OxfordOxford, UK

**Keywords:** apoptosis, negative feedback, SHP1, TCR, THEMIS

## Abstract

THEMIS is critical for conventional T-cell development, but its precise molecular function remains elusive. Here, we show that THEMIS constitutively associates with the phosphatases SHP1 and SHP2. This complex requires the adapter GRB2, which bridges SHP to THEMIS in a Tyr-phosphorylation-independent fashion. Rather, SHP1 and THEMIS engage with the N-SH3 and C-SH3 domains of GRB2, respectively, a configuration that allows GRB2-SH2 to recruit the complex onto LAT. Consistent with THEMIS-mediated recruitment of SHP to the TCR signalosome, THEMIS knock-down increased TCR-induced CD3-ζ phosphorylation, Erk activation and CD69 expression, but not LCK phosphorylation. This generalized TCR signalling increase led to augmented apoptosis, a phenotype mirrored by SHP1 knock-down. Remarkably, a KI mutation of LCK Ser59, previously suggested to be key in ERK-mediated resistance towards SHP1 negative feedback, did not affect TCR signalling nor ligand discrimination *in vivo*. Thus, the THEMIS:SHP complex dampens early TCR signalling by a previously unknown molecular mechanism that favours T-cell survival. We discuss possible implications of this mechanism in modulating TCR output signals towards conventional T-cell development and differentiation.

## Introduction

Cell fate choices are epitomized by death-mediated cell disposal, survival, lineage commitment, proliferation and differentiation. Regulatory feedback and feed-forward mechanisms tailor signal kinetics and amplitude to translate signal inputs into stereotypical gene expression patterns associated with diverse cell fates (Alon, 2007). T cells represent an outstanding example of this paradigm, being capable of translating different incoming T-cell antigen receptor (TCR) signals into many diverse biological outcomes (Gascoigne & Palmer, 2011). A distinctive feature of TCR signalling is its sensitivity to low doses of agonist ligands (peptide/MHC, pMHC) of relatively weak affinity, combined with the capability to subtly discriminate between signals induced by ligands of close affinity(Gascoigne & Palmer, 2011). Consequently, fine differences in analogue input signals can be converted, at set thresholds, into digital outputs, which can ultimately lead to opposing or divergent outcomes (Daniels *et al*, 2006; Naeher *et al*, 2007). The convoluted modular assembly of the TCR signalling machine controlled by sequential phosphorylation events and regulatory mechanisms provides the molecular framework for accurately and unfailingly executing these subtle tasks (Altan-Bonnet & Germain, 2005; Acuto *et al*, 2008). However, the molecular circuits driving these choices in different developmental and functional contexts such as in thymocytes and mature T cells are far from being understood (Acuto *et al*, 2008).

THEMIS, a recent addition to the TCR-proximal signalling machine (Brockmeyer *et al*, 2011; Paster *et al*, 2013; Fu *et al*, 2014), is rapidly tyrosine-phosphorylated upon TCR ligation and is required for the differentiation of immature CD4/CD8 double-positive (DP) thymocytes into mature CD4 or CD8 single-positive (SP) thymocytes (Fu *et al*, 2009; Johnson *et al*, 2009; Kakugawa *et al*, 2009; Lesourne *et al*, 2009; Patrick *et al*, 2009). THEMIS is expressed only in the T-cell lineage and is first detected in CD4/CD8 double-negative (DN) thymocytes, reaching maximum levels at the DP stage. Following thymocyte selection, THEMIS expression is decreased in mature CD4 and CD8 SP thymocytes and peripheral T cells. Remarkably, while THEMIS is still well expressed in conventional T cells (*T*_conv_), it is barely detected or absent in regulatory T cells (*T*_reg_) and intestinal CD8αα IELs (Johnson *et al*, 2009; Lesourne *et al*, 2009). Thus, THEMIS appears to be important for *T*_conv_ development (Fu *et al*, 2013) and may as well play a role in mature, peripheral *T*_conv_. We have shown that soon after TCR stimulation, THEMIS associates to LAT, via the adapter protein GRB2, leading to THEMIS phosphorylation, and that this recruitment is required for THEMIS to function in *T*_conv_ development (Paster *et al*, 2013). Despite the severe phenotype of THEMIS-deficient mice and initial hints that THEMIS was implicated in regulating TCR signalling (Fu *et al*, 2009; Brockmeyer *et al*, 2011), its precise molecular and functional role has remained obscure.

In this work, we used mass spectrometry (MS) to search for THEMIS binding partners. We found that in addition to GRB2, THEMIS was also associated with the tyrosine phosphatase SHP1 (PTPN6) and SHP2 (PTPN11). SHP1 and SHP2 constitutively interacted with THEMIS, in a GRB2-dependent fashion. We describe a previously unreported binding of SHP1 to GRB2 N-SH3, so that GRB2 C-SH3 bridges to THEMIS and leaves GRB2 SH2 available for recruiting the complex onto phosphorylated LAT. In agreement with a role of SHP1 in attenuating TCR-proximal signalling (Fawcett & Lorenz, 2005; Sankarshanan *et al*, 2007), we found that reducing levels of THEMIS by shRNA resulted in increased TCR-proximal signalling as demonstrated by augmented CD3-ζ and Erk activation. Moreover, increased apoptotic cell death was observed upon TCR stimulation in cells with reduced THEMIS expression. Consistently, shRNA-mediated knock-down of SHP1 also increased TCR-induced cell death, and we found no role for SHP1 tyrosine phosphorylation in this context. We also asked whether the function of the THEMIS:SHP complex in TCR signalling was akin to a previously proposed SHP-mediated negative feedback mechanism that relies on a direct interaction between LCK and SHP and is prevented when Ser59 of LCK is phosphorylated by Erk (Stefanova *et al*, 2003). However, we found no evidence for THEMIS:SHP association with LCK after TCR stimulation, nor an effect of THEMIS knock-down on LCK phosphorylation status. Moreover, a knock-in (KI) mutation of LCK-Ser59 was without effect on TCR signalling or ligand discrimination *in vivo*. These data together with our recent *in vivo* evidence that THEMIS is key for setting the threshold between positive and negative selection of conventional T cells (Fu *et al*, 2013) support the physiological relevance of the THEMIS:GRB2:SHP1 complex as a novel mediator of a negative feedback mechanism that modulates TCR phosphorylation to favour T-cell development and activation.

## Results

### THEMIS constitutively interacts with SHP proteins in a GRB2-dependent manner

**Figure 1:**
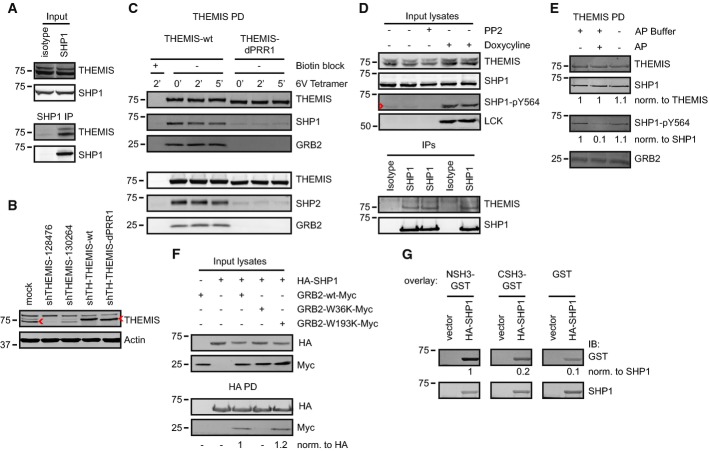
SHP1 and SHP2 binding to THEMIS is GRB2 dependent
SHP1 immunoprecipitation (IP) from Jurkat 1G4-CD8 cells. Upper panel of immunoblots shows expression levels in the input lysates, and isolated protein complexes are shown at the bottom. Rabbit IgG IP (isotype) is shown as control.Lentiviral knock-down/re-expression constructs for THEMIS-wt-Strep and THEMIS-dPRR1-Strep in 1G4-CD8 cells. Knock-down/re-expression constructs are based on shTHEMIS-128476. Red arrowheads indicate the specific signal for endogenous THEMIS and re-expressed THEMIS-Strep. The higher molecular weight band observed in all lanes is a non-specific signal.Streptactin pull-downs (PDs) of THEMIS-Strep from 1G4-CD8 cells. Cells as described in (B) were stimulated with 6V tetramers, followed by Streptactin PDs of THEMIS and immunoblot analysis of isolated protein complexes. Separate experiments for SHP1 and SHP2 are shown.SHP1 IP in the presence and absence of SHP1 tail phosphorylation. SHP1 was immunoprecipitated from J.CaM1.6 LCK-Tet cells. Where indicated, LCK expression was induced with doxycycline prior to the experiment or Src-kinase inhibitor PP2 was used to abolish residual SHP1-pY564. The upper panel of immunoblots shows the effect of LCK activity on SHP1-pY564 levels. The red arrowhead indicates residual pY564. Isolated protein complexes are shown in the lower panels.*In vitro* phosphatase treatment of the THEMIS:GRB2:SHP1 complex. Bead-bound THEMIS-Strep complexes from 1G4-CD8 cells were incubated in alkaline phosphatase (AP) buffer in the absence (lane 1) or presence of AP (lane 2), or left completely untreated (lane 3), prior to washing and elution.Anti-HA IP from HEK293 cells transfected with HA-SHP1 and GRB2-Myc constructs. GRB2 mutants used: W36K, N-SH3 mutant; W193K, C-SH3 mutant. Upper panels show expression levels in the input; isolated proteins complexes are shown at the bottom. Relative amounts of GRB2-Myc are normalized to the bait HA-SHP1.Far-Western blot of GRB2-SH3 domains binding to full-length SHP1. HA IPs from empty vector or HA-SHP1-transfected HEK293 cells were subjected to far-Western blotting using recombinant GST-tagged N -or C-SH3 domains of GRB2. GST alone served as a background control. Blots were re-probed for SHP1 loading for normalization of anti-GST signals. Data shown are representative of three independent experiments.

To gain insight into THEMIS function, we carried out MS-based identification of its protein-binding partners. THEMIS tagged with One-STrEP-Tag (THEMIS-Strep) was expressed in Jurkat cells and pulled down with Streptactin beads from either resting or anti-CD3 mAb-stimulated cells. Isolated protein complexes were eluted with biotin and analysed by nanoLC-MS/MS. In agreement with published data, in resting cells, THEMIS co-purified with GRB2 (Table1 and Supplementary Table S1) (Johnson *et al*, 2009; Lesourne *et al*, 2009; Brockmeyer *et al*, 2011; Paster *et al*, 2013). However, ranking just after GRB2 for similar sequence coverage and spectral counts (SC) (see, Table1, Supplementary Table S1 and Materials and Methods), we found the protein tyrosine phosphatases (PTPs) SHP1 (PTPN6) and SHP2 (PTPN11) to be >20-fold enriched over background in both non-stimulated and stimulated cells. Apart from GRB2, SHP1 and SHP2, no other protein was comparably detected or enriched over background in subsequent MS analyses. Because SHP1 and SHP2 share significant structural and functional homology (Lorenz, 2009), and both co-purified with THEMIS, we inferred that they might be genuine THEMIS functional partners. These data were corroborated by SHP1 immunoprecipitation experiments from CD8^+^ Jurkat 1G4 cells (Jurkat 1G4-CD8 hereafter, Fig1A). Jurkat 1G4-CD8 express an αβ TCR specific for an HLA-A2-restricted NY-ESO-1 cancer–testis peptide (Davis *et al*, 2012). The 1G4-CD8 model and the NY-ESO-1 peptide variants (Aleksic *et al*, 2010) used throughout this work are introduced in >Supplementary Fig S2A. To investigate the THEMIS:SHP complex in greater detail, an shRNA-resistant mutant of THEMIS-Strep was co-expressed with an shTHEMIS hairpin in Jurkat 1G4-CD8, effectively replacing endogenous THEMIS with THEMIS-Strep (see Materials and Methods). Both wt THEMIS and a deletion mutant of the proline-rich region (dPRR1), which cannot associate with GRB2 (Paster *et al*, 2013), were expressed in this knock-down/re-expression system (Fig1B). THEMIS-Strep pull-down showed a preformed complex including GRB2 and both SHP1 and SHP2 (Fig1C). TCR stimulation by NY-ESO-1 6V peptide/HLA-A2 tetramer did not affect complex formation. However, THEMIS-dPRR1 failed to bind to both SHP1 and SHP2 (Fig1C). Similarly, the potentially less disruptive THEMIS-R555A point mutant, specifically targeting a key residue in the PxRPxK-motif within PRR1 of THEMIS (Paster *et al*, 2013), no longer bound both GRB2 and SHP1 (Supplementary Fig S1A). Together, these data suggested that T cells express at steady-state THEMIS:GRB2:SHP complexes, whose composition appears to remain unchanged upon TCR stimulation.

**Table 1:** THEMIS interactors indentified by mass spectrometry.

Accession^a^	Peptide sequences	Sequence coverage (%)	SC resting	SC 2 min UCHT-1	SC biotin block
Q8N1K5|THMS1_HUMAN	41	59.60	88.16	113.9	–
P62993|GRB2_HUMAN	9	44.70	8.96	9.94	–
Q06124-2|PTN11_HUMAN	7	15.20	2	4	–
P29350-3|PTN6_HUMAN	5	9.20	3.96	1.98	–
P31146|COR1A_HUMAN	3	6.30	2	–	–
Q9NPA3|M1IP1_HUMAN	3	14.80	–	2	–

a Only proteins >20-fold over biotin-block control and filtered via CRAPOME (www.crapome.org) are shown.

For details see Materials and Methods.

### SHP tyrosine phosphorylation in T cells is dispensable for GRB2-mediated THEMIS:SHP complex formation

The tyrosines in the C-terminal regulatory region of SHP1 and SHP2 (Tyr536/Tyr564 and Tyr542/Tyr580, respectively) (Bennett *et al*, 1994; Minoo *et al*, 2004) are thought to play a role in the regulation of phosphatase activity and in providing adapter function via binding to SH2-domain-containing proteins (Bennett *et al*, 1994; Lu *et al*, 2001; Zhang *et al*, 2003). For instance, using those phosphorylated residues, the SHP enzymes can associate with the SH2 domain of GRB2 (Bennett *et al*, 1994; Minoo *et al*, 2004). Our recent work found that GRB2 associates constitutively via its C-SH3 domain to the PxRPxK-motif in the C-terminus of THEMIS (Paster *et al*, 2013). Thus, the interaction between SHP1 and GRB2 might be driven by SHP1 C-terminal Tyr-phosphorylation and GRB2-SH2. Indeed, in 1G4-CD8 (Supplementary Fig S1B) and normal T cells (Supplementary Fig S1C), pTyr564-SHP1 was detected at steady state. Quantitative pull-down of THEMIS-Strep (∽90% of cellular pool) contained pTyr564-SHP1, which remained unchanged upon TCR stimulation (Supplementary Fig S1B). However, approximately < 1% of pTyr564-SHP1 species is bound to THEMIS (Supplementary Fig S1B), leading us to question the implication of the SHP C-terminal tyrosines in the formation of the THEMIS:SHP complex. Because SHP1 C-terminal Tyr-phosphorylation is LCK dependent [(Lorenz *et al*, 1994) and see below], we initially inhibited LCK activity with the Src-kinase inhibitor PP2 in 1G4-CD8 cells expressing THEMIS-Strep (Supplementary Fig S1D). PP2 treatment markedly decreased LCK autophosphorylation on Tyr394 and reduced 70% of pTyr564-SHP1. However, no change in the amount of SHP1 was detected following THEMIS-Strep pull-down. To completely rule out that residual C-terminal Tyr-phosphorylation might still ensure THEMIS:GRB2:SHP1 complex formation, we turned to the LCK-deficient Jurkat clone J.CaM1.6 (Fig1D). JCaM1.6 showed poor basal phosphorylation of Tyr564 of SHP1, which became undetectable after PP2 treatment, and yet no change in the amounts of SHP1 associated with THEMIS was noticed. Re-expression of LCK resulted in robust Tyr564 phosphorylation with no appreciable change in the stoichiometry of SHP1 bound to THEMIS. Finally, the THEMIS:GRB2:SHP complex was captured on beads, treated with alkaline phosphatase (AP), which reduced Tyr564 phosphorylation by 90% and washed before elution (Fig1E). This experiment showed again no detectable change in the amounts of GRB2 and SHP1 bound to THEMIS. Together, these data provide strong evidence that in the context of SHP1 association with THEMIS, the GRB2 SH2 domain does not participate in bridging these partners.

### GRB2 SH3 domains bridge THEMIS to SHP

We therefore tested which of the GRB2 SH3 domains was involved in SHP binding. HA-immunoprecipitations were performed from HEK293 cells transiently co-transfected with HA-SHP1 and GRB2-Myc wt, or mutants of the N-SH3 (W36K) and C-SH3 domains (W193K) (Bisson *et al*, 2011). Whereas wt GRB2 bound to SHP1, the W36K mutant drastically reduced SHP1 interaction (Fig1F). However, the W193K mutation had no effect on SHP1 binding. To ascertain whether N-SH3-mediated GRB2 binding to SHP proteins was direct or indirect, we carried out far-Western experiments using recombinant GRB2 SH3 GST fusion proteins on HA-SHP1 isolated from transfected HEK293 cells (Fig1G). Recombinant GRB2 N-SH3-GST recognized SHP1, whereas GRB2 C-SH3-GST did so only poorly and at the level of the GST-only control. Together, these data suggested that in T cells, GRB2 bridges THEMIS and SHP1, exploiting both of its SH3 domains each bound to one partner. Consequently, GRB2-SH2 will remain unoccupied, allowing THEMIS recruitment to phosphorylated GRB2-binding sites on LAT after TCR stimulation, as we have previously shown (Paster *et al*, 2013).

### THEMIS deficiency augments TCR-proximal signalling

**Figure 2:**
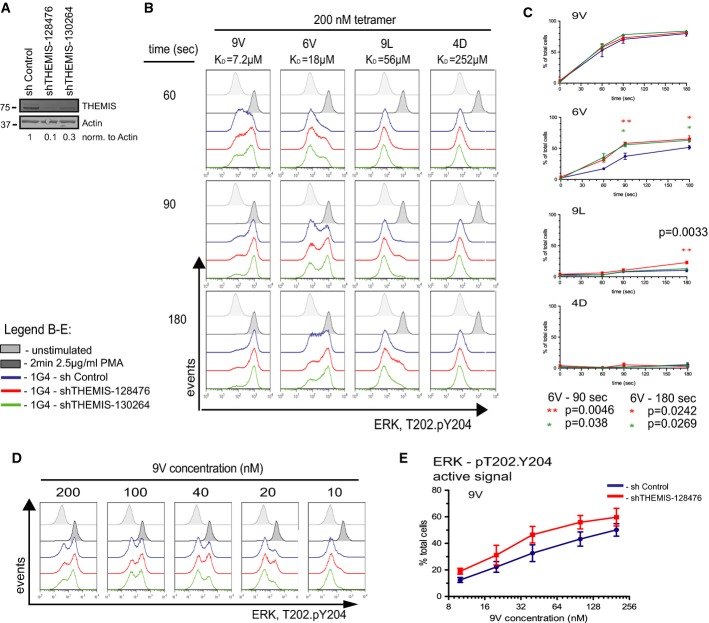
THEMIS knock-down increases TCR-induced phospho-ERK in Jurkat 1G4-CD8 cells
Lentiviral knock-down (KD) of THEMIS expression in 1G4-CD8 cells. The efficiency of the THEMIS KD was assessed using immunoblotting.Phospho-flow analysis of pERK responses in THEMIS KD cells. 1G4-CD8 control and THEMIS KD cells were stimulated with NY-ESO-1 pMHC tetramers and analysed for ERK phosphorylation using flow cytometry. Data shown are representative of three independent experiments.Quantification of pERK-positive cells from (B). Data from three independent experiments were used in the analysis. *n* = 3, means ± SEM are shown; two-tailed unpaired Student's *t*-test, *P < 0.05, **P < 0.01.Phospho-flow analysis of pERK responses against titrated concentrations of 9V tetramer. Time point was fixed at 60 s. Data shown are representative of three independent experiments.Quantification of pERK-positive cells from (D). Data from three independent experiments were used in the analysis. *n* = 3, means ± SEM are shown.

Earlier *in vivo* studies using transgenic mouse models have implicated SHP1 in negative regulation of TCR signalling and thymus selection processes (Carter *et al*, 1999; Johnson *et al*, 1999; Plas *et al*, 1999; Zhang *et al*, 1999). The role of SHP2 in T cells remains less defined, though it has been suggested to serve both negative and positive regulatory functions (Qu *et al*, 2001; Nguyen *et al*, 2006). SHP1 appears to be capable of dephosphorylating TCR-proximal signalling components (Plas *et al*, 1996; Binstadt *et al*, 1998; Stefanova *et al*, 2003). Because THEMIS is recruited onto LAT (Paster *et al*, 2013) and is constitutively associated with SHP1, we hypothesized that the latter could be transported near the TCR signalling machinery upon TCR ligation and down-modulate the incoming signal. To test this hypothesis, we made use of Jurkat 1G4-CD8 cells stimulated with HLA-A2 tetramers presenting the NY-ESO-1 peptide 9V (*K*_D_ = 7.2 μM) or, ranked by decreasing affinity for 1G4 TCR, single amino acid-substituted NY-ESO-1 variants 6V (*K*_D_ = 18 μM), 9L (*K*_D_ = 56 μM) and 4D (*K*_D_ = 252 μM) (Supplementary Fig S2A). We validated this experimental system by monitoring Erk activation by flow cytometry. Supplementary Figure S2B shows FACS plots of the kinetics of TCR occupancy by each of the four tetramers and the corresponding effect on Erk activation (pT202/pY204-Erk). As expected, stimulation reveals a digital response for Erk activation (Altan-Bonnet & Germain, 2005; Daniels *et al*, 2006). The data showed that binding kinetics and maximal TCR occupancy correlated well with Erk activation and this was in accordance with increased tetramer affinity (Supplementary Fig S2C and D). This experimental system using genuine TCR ligands was therefore suitable for assessing TCR signalling phenotypes caused by genetic manipulation. To test the effect of THEMIS deficiency on TCR signalling, we employed two lentivirus-based shRNA constructs against human THEMIS, achieving routinely 70 and 90% knock-down (KD) efficiency in 1G4-CD8 cells (Fig2A). Fig2B shows a representative kinetics experiment (60–180 s) of pErk induction following stimulation by 9V, 6V, 9L and 4D tetramers of 1G4-CD8 THEMIS KD and control cells. Figure2C summarizes the results of multiple experiments. Whereas the response of THEMIS KD cell lines was indistinguishable from control cells when stimulated by 9V, with 6V stimulation, both KD cell lines showed a significantly higher response over the control cell line. Moreover, with 9L tetramer, a significant increase of pErk compared to control was seen in the 90% THEMIS KD cell line. Although not reaching statistical significance, the pERK response of 90% THEMIS KD cells towards decreasing doses of 9V (from 200 to 10 nM) was consistently elevated when compared to control cells (Fig2D and E).

**Figure 3:**
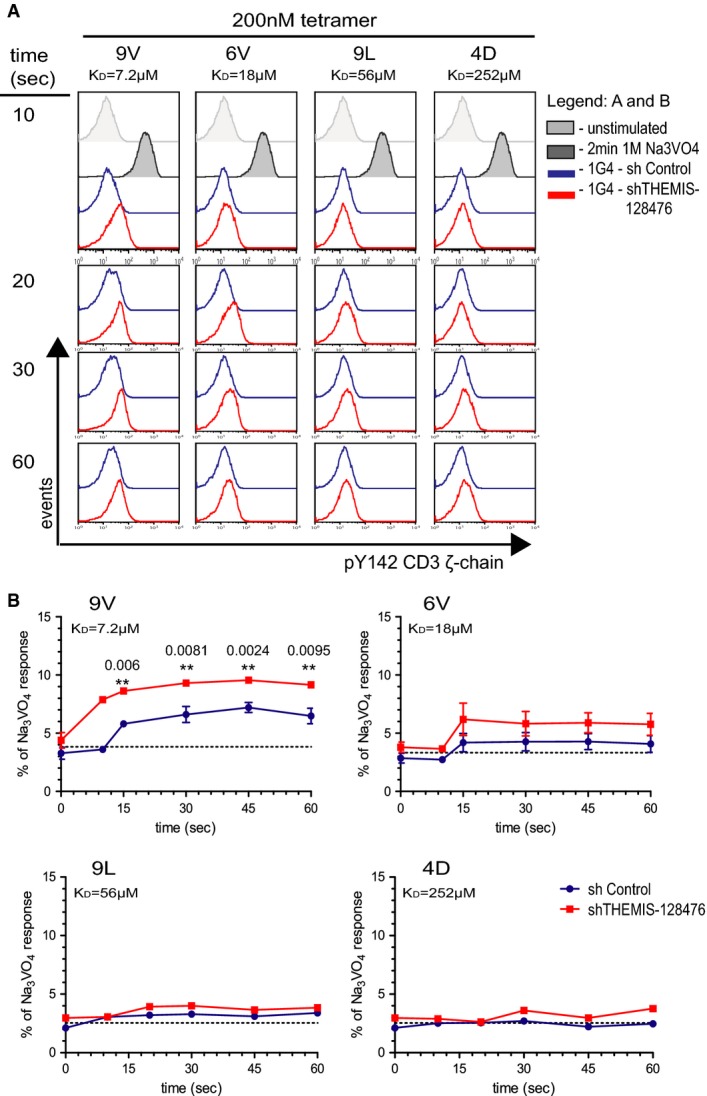
THEMIS knock-down increases TCR-induced pY142-CD3-ζ phosphorylation
1G4-CD8 control and THEMIS KD cells were stimulated with NY-ESO-1 pMHC tetramers and analysed for pY142-CD3-ζ phosphorylation by flow cytometry. Data shown are representative of three independent experiments.Quantification of pY142-CD3-ζ responses of cells from (A). Data from three independent experiments were used in the analysis. Responses are expressed as percentage of the positive control sodium pervanadate. *n* = 3, means ± SEM are shown; two-tailed unpaired Student's *t*-test, **P < 0.01.

**Figure 4:**
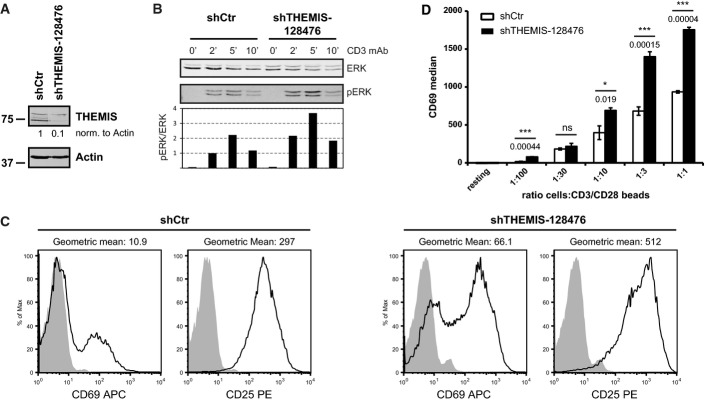
THEMIS knock-down increases TCR signalling in peripheral CD4^+^ T cells
Lentiviral knock-down of THEMIS expression in primary human CD4^+^ T cells. Following transduction, cells were expanded on CD3/CD28 beads in the presence of exogenous IL-2 and analysed for THEMIS KD by immunoblotting on day 4 post-transduction. Data shown are representative of three independent experiments.pERK response in THEMIS KD human CD4^+^ T cells. Transduced CD4^+^ cells were removed from beads, rested overnight and stimulated with CD3 mAb for the indicated time points. ERK phosphorylation was assessed by immunoblotting. Data shown are representative of three independent experiments.Expression of CD25 and CD69 surface markers of THEMIS KD human CD4^+^ T cells was assessed on day 4 post-transduction by flow cytometry. Data shown are representative of three independent experiments.CD69 up-regulation on re-stimulated THEMIS KD CD4^+^ T cells. CD3/CD28 beads were removed on day 1 post-transduction, and cells were cultured in IL-2 and IL-7. After removal of cell debris at day 4, cells were restimulated with CD3/CD28 beads at the indicated bead to cell ratio. CD69 surface expression was assessed by flow cytometry 24 h later. Data shown are representative of two independent experiments. *n* = 3, means ± SD are shown; two-tailed unpaired Student's *t*-test, *P < 0.05, ***P < 0.001.

These data revealed that THEMIS exerted a negative effect on the TCR signalling machinery, which might be due to the action of its binding partner SHP1 (and/or SHP2). To address the question of which of the multiple biochemical steps of the TCR signalling cascade were affected by THEMIS knock-down, we determined whether reduction of THEMIS expression influenced basal LCK activity and TCR-induced ζ-ITAM phosphorylation. Monitoring LCK-pY394 by immunoblot did not reveal convincing evidence for alterations in LCK activity in 90% THEMIS KD cells (Supplementary Fig S3A). However, detection of ζ-ITAM phosphorylation (pY142, Fig3A) showed a significant increase in 90% THEMIS KD cells as compared to control cells in response to 9V tetramer (Fig3B). A similar tendency was also observed when stimulated with 6V tetramer (Fig3B). Moreover, 9V tetramer titration experiments showed an increase of phospho-ζ in 90% THEMIS KD over control cells (Supplementary Fig S4A and B). These data were corroborated by cell-tracker dye experiments in which the 90% THEMIS KD and control lines were mixed, stimulated with 9V tetramer and analysed by FACS for ζ-ITAM phosphorylation (Supplementary Fig S4C–E). A similar increase in pErk activation was observed in THEMIS KD primary human CD4 T cells (Fig4A and B). These data suggest that the recruitment of the THEMIS:GRB2:SHP complex onto LAT evokes a negative feedback mechanism that reduces TCR signalling, thus curtailing the signalling cascade from its inception.

### THEMIS deficiency results in augmented T-cell activation markers and apoptotic cell death

**Figure 5:**
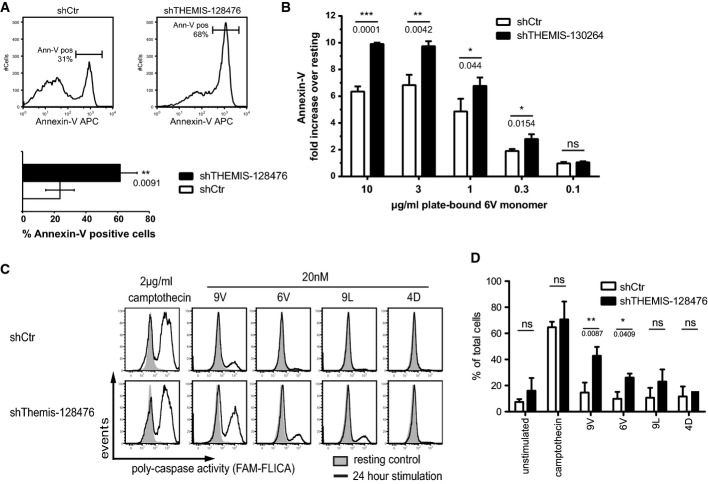
Increased TCR-induced apoptosis in THEMIS KD T cells
Donor-derived human CD4^+^ T cells transduced as in Fig4A were analysed for apoptosis by Annexin-V surface staining on day 4 post-transduction. Data from three independent experiments were used in the analysis. *n* = 3, means ± SD are shown; two-tailed unpaired Student's *t*-test, **P < 0.01.Annexin-V staining of THEMIS KD 1G4-CD8 cells. THEMIS KD and control 1G4-CD8 cells were stimulated for 24 h with plate-bound 6V tetramers. Annexin-V surface expression was analysed by flow cytometry and is shown as fold-increase over non-stimulated. Data from three independent experiments were used in the analysis. *n* = 3, means ± SD are shown; two-tailed unpaired Student's *t*-test, *P < 0.05, **P < 0.01, ***P < 0.001; ns, not significant.Poly-caspase activity in THEMIS KD 1G4-CD8 cells. Cells were stimulated for 24 h with NY-ESO-1 pMHC tetramers. Camptothecin was used as a positive control for caspase activation. A FAM-FLICA detection probe was used to assess poly-caspase activity by flow cytometry. Data shown are a representative example of three independent experiments.Quantification of poly-caspase activity of cells from (C). Data from three independent experiments were used in the analysis. *n* = 3, means ± SEM are shown; two-tailed unpaired Student's *t*-test, *P < 0.05, **P < 0.01; ns, not significant.

**Figure 6:**
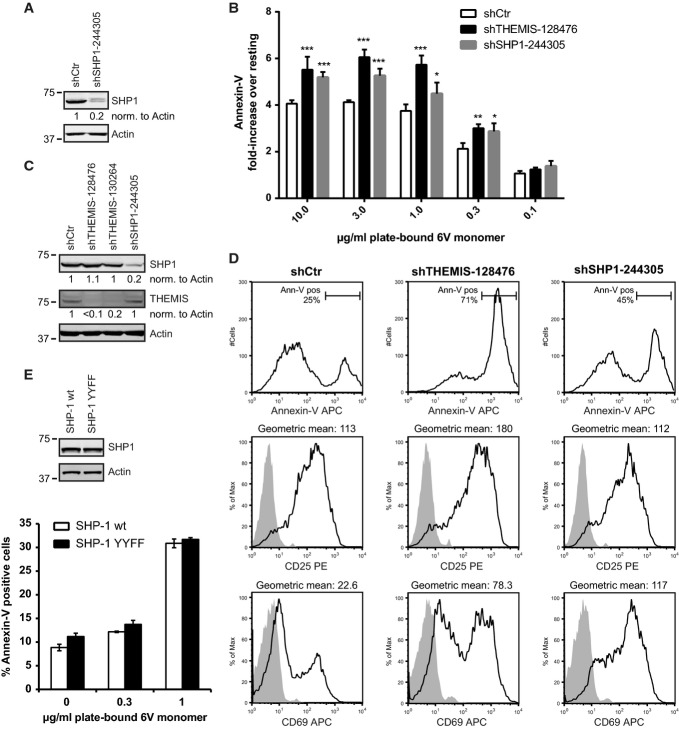
Increased TCR-induced apoptosis in SHP1 KD T cells
Lentiviral KD of SHP1 expression in 1G4-CD8 cells. KD efficiency was assessed using immunoblotting.Annexin-V staining of THEMIS and SHP1 KD 1G4-CD8 cells. Control, THEMIS and SHP1 knock-down Jurkat 1G4-CD8 cells were stimulated for 24 h with plate-bound 6V tetramers. Annexin-V surface expression was analysed by flow cytometry and is shown as fold-increase over non-stimulated. Data from three independent experiments were used in the analysis. *n* = 3, means ± SD are shown; two-way ANOVA, Bonferroni post-test, *P < 0.05, **P < 0.01, ***P < 0.001.Lentiviral knock-down of THEMIS and SHP1 expression in primary human CD4^+^ T cells. Following transduction, cells were expanded on CD3/CD28 beads in the presence of exogenous IL-2. Knock-down efficiency was analysed by immunoblotting on day 4 post-transduction.Expression of CD25, CD69 and Annexin-V surface markers of cells from (C) was assessed by flow cytometry. Data shown are representative of three independent experiments.Annexin-V staining of 1G4-CD8 cells re-expressing a SHP1 tail tyrosine mutant. Jurkat 1G4-CD8 cells were transduced with Knock-down/re-expression constructs for HA-SHP1 wild-type (wt) or Tyr536/564 to Phe double mutant (YYFF). Cells were stimulated and analysed by flow cytometry as in (B). Data shown are representative of three independent experiments. *n* = 3, means ± SD are shown.

Increased TCR-proximal signalling, as a consequence of THEMIS KD, should potentially result in augmented cell surface activation markers. We carried out experiments addressing this question in human CD4 T cells. Freshly isolated donor-derived human CD4 T cells were transduced with lentiviral THEMIS or control shRNA constructs. Cells were then expanded with anti-CD3/CD28-coated beads and rIL-2 and selected with puromycin. Consistent with increased TCR-proximal signalling and Erk activation (Fig4B), THEMIS KD in CD4 T cells lead to increased expression of both CD69 and CD25 as compared to control cells (Fig4C). The enhanced activation (e.g., CD69 expression) in THEMIS KD T cells was confirmed across a wide range of CD3/CD28 stimulatory strength (Fig4D). Interestingly, we noticed that cultures of THEMIS KD CD4 T cells (and to a lesser extent also 1G4 Jurkat cells) displayed a consistently higher rate of cell death when compared to control cells. As a consequence, prolonged stimulation of human T cells with anti-CD3/CD28 led to poor recovery of live cells in THEMIS KD cultures after several days. We therefore tested whether, in addition to increased proximal signalling, THEMIS deficiency also resulted in augmented cell death. Consistent with this hypothesis, increased apoptosis was observed in CD3/CD28-stimulated THEMIS KD human CD4 T cells as detected by Annexin-V staining (Figs5A and 6D). Activation-induced cell death (AICD) is mediated by the interaction of death factors and their receptors and plays an important role in immune system homeostasis. The Fas-FasL system is important for AICD in peripheral T cells, and differential expression levels could modulate sensitivity to AICD (Krammer *et al*, 2007). No increase of Fas (CD95) or its ligand FasL (CD178) on the cell surface was observed at the time of Annexin-V staining (Supplementary Fig S5A). Moreover, THEMIS KD 1G4-CD8 cells stimulated with plate-bound NY-ESO-1 6V MHC tetramer showed a clear increase in Annexin-V staining when compared to control cells (Fig5B). To corroborate these data, THEMIS KD and control 1G4-CD8 cells were subjected to stimulation by NY-ESO-1 9V, 6V, 9L and 4D MHC tetramers and analysed for poly-caspase activation by flow cytometry. Figure5C and D shows that THEMIS KD resulted in significantly augmented TCR-induced caspase activation. As for human T cells, THEMIS KD and control cells expressed identical surface levels of CD95 and CD178 under both resting and tetramer stimulated conditions (Supplementary Fig S5B). These data indicated that THEMIS KD leads to increased TCR-induced cell death correlating with augmented TCR-proximal signalling. Thus, by negatively regulating TCR-proximal signalling, THEMIS limits activation-induced apoptotic cell death.

### Reduced SHP1 expression increases TCR-induced apoptosis

The *motheaten*(*me*) mutant mouse strain (SHP1 null mutant) shows a severe autoimmune and immunodeficiency syndrome and exhibits a strong defect in T-cell maturation (Lorenz, 2009). This phenotype is likely to be exacerbated by a combined defect in the innate immune compartment, which is also affected by SHP1 deficiency (Pao *et al*, 2007). However, transgenic over-expression of a SHP1 loss-of-function mutant in otherwise normal mice enhanced both positive and negative selection (Plas *et al*, 1999; Zhang *et al*, 1999). It is unclear whether increased activation-induced T-cell death can be caused by SHP1 deficiency. Our findings that THEMIS constitutively associated with both SHP1 and SHP2 suggest that they could be both recruited onto LAT (Paster *et al*, 2013), thus initiating negative feedback that attenuates TCR signalling. To test this hypothesis, SHP1 was silenced in 1G4-CD8 cells by a lentiviral shRNA construct. An 80% reduction of SHP1 expression in 1G4-CD8 cells (Fig6A) led to a significant increase in Annexin-V-positive cells after stimulation with plate-bound NY-ESO-1 6V MHC tetramer as compared to control cells, an effect similar to that observed for THEMIS KD cells (Fig6B). Moreover, SHP1 KD primary human T cells displayed an increase of CD69 and Annexin-V-positive cells following CD3/CD28 stimulation when compared to control cells, reminiscent of the THEMIS KD phenotype (Fig6D). These data suggest that SHP1 mediates negative feedback after TCR stimulation and is able to reduce T-cell death. The similar effect of THEMIS and SHP1 on cell death and their physical association shown here strongly supports the notion that the function of the THEMIS:GRB2:SHP1 complex is to reduce cell death during T-cell activation.

SHP1 phosphorylation at Tyr536 and Tyr564 did not appear to play a role in THEMIS-mediated negative regulation of TCR signalling, as the replacement of SHP1 wt for a SHP1 Tyr536/564 to Phe double mutant had no effect on TCR-induced cell death (Fig6E). Attempts to determine whether SHP1 N-SH2 and C-SH2 domains played a role in the regulation of SHP1 activity in the context of THEMIS association were thwarted by considerably reduced expression levels of the corresponding mutants, likely due to the mutations affecting protein stability.

### Mutation of Ser59 of LCK does not affect TCR signalling and ligand discrimination *in vivo*

It has been suggested that upon recognition of strong agonist ligands by the TCR, LCK undergoes Ser phosphorylation at position 59, preventing recruitment of SHP1 and subsequent TCR desensitization (Stefanova *et al*, 2003). Such a positive feedback loop would be inefficiently induced by weak agonist ligands and therefore thought to increase TCR ligand discrimination (Altan-Bonnet & Germain, 2005). To address the importance of LCK-Ser59 *in vivo*, we generated knock-in mice, in which Ser59 was replaced by Ala (Supplementary Fig S6A and B). Mice homozygous for this mutation, *Lck*^S59A^ mice, were born at expected Mendelian frequencies, and T-cell expression of LCK^S59A^ was comparable to wild-type LCK (Supplementary Fig S6C). Analysis of *Lck*^S59A^ mice expressing a polyclonal TCR repertoire showed that their thymi and secondary lymphoid organs were of normal size and cellular composition (Supplementary Fig S6D and E). Monitoring CD4, CD8, CD44, CD25, CD3 and CD5 expression showed no major alteration in T-cell populations from thymus and secondary lymphoid organs (Supplementary Fig S6D and E). T-cell proliferation and IL-2 and IFN-γ production in response to graded concentrations of anti-CD3 antibody, or to a suboptimal dose of anti-CD3 antibody in combination with increasing concentrations of anti-CD28 antibody, were similar in *Lck*^S59A^ and wild-type mice.

**Figure 7:**
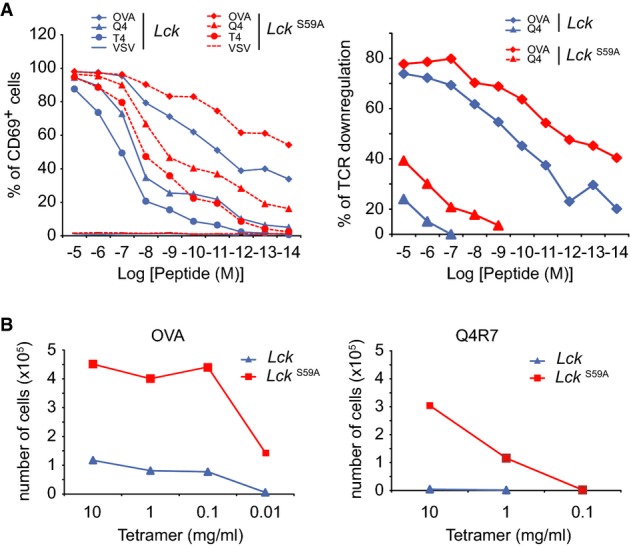
T-cell activation in OT-I TCR transgenic mice expressing LCK^S^^59A^ molecules
CD8^+^ T cells from OT-I *Rag2*^−/−^ mice expressing wild-type (LCK) or LCK^S^^59A^ proteins were stimulated with H-2K^b^-positive antigen-presenting cells pulsed with serial dilutions of the OVA (257–264) peptide, the OVA peptide variants Q4 and T4, and VSV, a peptide that is not recognized by the OT-I TCR. CD69 up-regulation (left) and TCR down-regulation (right) were analysed. Data are representative of three independent experiments.CD8^+^ T cells from OT-I *Rag2*^−/−^ mice expressing wild-type (LCK) or LCK^S^^59A^ proteins were labelled with CFSE and stimulated with serial dilution of H-2K^b^-OVA or H-2K^b^-Q4R7 peptide-MHC tetramers. After 3 days, the absolute numbers of divided T cells were determined. Data are representative of three independent experiments.

To analyse with a higher sensitivity the effect of the *Lck*^S59A^ mutation on T-cell development and TCR ligand discrimination, *Lck*^S59A^ mice were backcrossed onto OT-I mice expressing a TCR specific for an ovalbumin peptide (OVA257-264). Tracking the monoclonal population of Vα2^+^ OT-I CD8^+^ T cells that develops in OT-I *Lck*^S59A^*Rag2*^−/−^mice showed that the presence of LCK^S59A^ had no effect on T-cell development (Supplementary Fig S7A and B). Moreover, normal numbers of Vα2^+^ OT-I CD8^+^ T cells were found in secondary lymphoid organs (Fig S7C). CD69 up-regulation has been used to measure the potency of variants of the OVA257-264 peptide denoted as Q4, T4 and Q4R7 (Daniels *et al*, 2006). The presence of LCK^S59A^ molecules did not compromise CD69 up-regulation in responses to the OVA agonist and the Q4 and T4 weak agonists and slightly better responses were observed for all peptides as compared to those of OT-I T cells expressing wild-type LCK molecules (Fig7A). Likewise, OVA and Q4 induced the down-modulation of the OT-I TCR and had a slightly greater potency in the presence of LCK^S59A^ molecules (Fig7A). Finally, when stimulated with H-2K^b^-OVA or H-2K^b^-Q4R7 peptide-MHC tetramers, T cells from OT-I *Rag2*^−/−^ mice expressing LCK^S59A^ proteins showed a higher proliferation than those from OT-I *Rag2*^−/−^ mice expressing wild-type LCK proteins (Fig7B). Therefore, LCK^S59A^ did not compromise TCR signalling in response to agonist and weak agonist ligands and it even led to slightly enhanced T-cell responses. Importantly, the potency hierarchy for all the tested OVA variant peptides was fully preserved when measured in the presence of LCK^S59A^ molecules, suggesting that Ser59 of LCK has a modest role in the SHP-based negative feedback pathway thought to implement TCR ligand discrimination.

In line with these data, we consistently failed to detect LCK in THEMIS:GRB2:SHP complexes, analysed by MS or immunoblotting (Supplementary Fig S1B). Additionally, inhibition of ERK activity in Jurkat 1G4-CD8 did not ameliorate apoptosis induced by plate-bound NY-ESO-1 pMHC tetramers (Supplementary Fig S8).

Altogether, these data suggest that the mechanistic model proposed by Germain and co-workers (Stefanova *et al*, 2003) to explain T-cell ligand discrimination is distinct from the one enacted by the THEMIS:GRB2:SHP1 complex (see Discussion).

## Discussion

We have shown that THEMIS is required to enact a TCR-induced negative feedback mechanism that modulates signal transmission. Previous work revealed similar TCR-induced negative feedback devices originating from the LAT:SLP-76 complex, which were mediated by the adaptors DOK-1/DOK-2 or the kinase HPK1 (Dong *et al*, 2006; Di Bartolo *et al*, 2007). However, in contrast to THEMIS KO mice, HPK1 KO or DOK-1/DOK-2 double KO showed no detectable defect of T-cell development (Shui *et al*, 2007; Yasuda *et al*, 2007). Thus, THEMIS is part of an as yet unique TCR-induced regulatory mechanism critical to drive T-cell development (Fu *et al*, 2013). We found that THEMIS is constitutively associated with SHP1 and SHP2 through the adaptor GRB2. THEMIS knock-down in human T cells and Jurkat 1G4-CD8 cells increased CD3-ζ phosphorylation and downstream activation events. Because TCR ligation induces recruitment of THEMIS, via GRB2, onto LAT (Paster *et al*, 2013), we infer that THEMIS allows transport of SHP1 and/or SHP2 close to ligand-activated TCRs, thus dampening TCR-ITAM phosphorylation and downstream signalling cascades. LAT deficiency also leads to augmented TCR-ITAM and ZAP-70 phosphorylation (Salek *et al*, 2013), further supporting the role of LAT as a hub for signal regulation (Acuto *et al*, 2008; Mingueneau *et al*, 2009; Shen *et al*, 2010). However, the *in vivo* consequences of LAT and THEMIS deficiency are substantially different (Acuto *et al*, 2008; Mingueneau *et al*, 2009; Fu *et al*, 2013; Shen *et al*, 2010), reflecting perhaps the capacity of the TCR to utilize additional signalling conduits that cannot tightly control the TCR signal as LAT does (Roncagalli *et al*, 2014).

THEMIS KD in CD4 T cells and 1G4 Jurkat cells had a higher propensity than control cells to die after TCR (and CD28, for primary CD4 T cells) stimulation. Increasing ligand affinity or concentration in Jurkat 1G4-CD8 cells directly correlated with increased apoptotic cell death. Previous work by Lenardo and co-workers has shown that robust TCR signalling leads to T-cell death (Combadiere *et al*, 1998). Therefore, it was not surprising that augmented TCR-proximal signalling, as a result of reduced THEMIS or SHP1 expression, increased T-cell death. This correlated with increased caspase activity but in the case of peripheral human T cells, it was not caused by IL-2 withdrawal, as this cytokine was exogenously added at high concentration to the cell cultures. Thus, it seems that a THEMIS:GRB2:SHP complex helps antagonize an early tendency of T cells to undergo TCR-induced apoptotic death during activation, by a cell-intrinsic mechanism likely mediated by BH3-only, Bcl-2 family members-/mitochondria stress-mediated mechanism (Krammer *et al*, 2007). An interesting corollary of our findings is that reduced transition of THEMIS-deficient DP to SP thymocytes (Fu *et al*, 2009) can be explained by the former cells undergoing cell death instead of survival, thus being negatively rather than positively selected. Consistent with this idea, in a recent work, we have observed that THEMIS-deficient DP thymocytes respond to positively selecting weak agonist pMHC ligands with increased TCR-induced signalling (e.g., LAT and PLCγ1 phosphorylation, [Ca^2+^]_*i*_ and Erk activation) and more pronounced apoptotic cell death (Fu *et al*, 2013). The THEMIS^−/−^ phenotype was almost completely rescued in the absence of the pro-apoptotic BH3-only, Bcl-2 family member, Bim. Moreover, in agreement with the data presented here, we found THEMIS associated with SHP1 also in thymocytes (SHP2 was not tested) (Fu *et al*, 2013). Combined, these results constitute a coherent ensemble of *in vitro* and *in vivo* evidence revealing a ‘signal dampening’ function enforced by THEMIS in both DP thymocytes and mature T cells. They provide a plausible explanation for an apparent ‘THEMIS deficiency puzzle’: a relatively mild (or hard to detect) TCR signalling phenotype leading to a severe ablation of *T*_conv_ development (Fu *et al*, 2009, 2013; Johnson *et al*, 2009; Lesourne *et al*, 2009).

We found that GRB2 engages its N-SH3 and C-SH3 domains to bind to SHP1 and THEMIS, respectively, thus linking them together and leaving the GRB2 SH2 domain free to interact with LAT (this work and Paster *et al*, 2013). Because quantitative *in cell* or *in vitro* dephosphorylation of SHP1 did not alter THEMIS:GRB2:SHP1 stoichiometry, we deduce that pTyr at the C-terminus of SHP1 does not play a major role in complex formation. Thus, while a detectable proportion of pTyr564-SHP1 at steady state might be associated with GRB2 via GRB-SH2, such a SHP1 pool could play a role in other signalling pathways, but not via association to THEMIS, to modulate TCR signalling. Moreover, a functional role of SHP1 C-terminal phosphorylation in regulating SHP1 activity in the context of the TCR-induced THEMIS-mediated negative feedback mechanism seems unlikely. Indeed, we did not observe changes in the amounts of pTyr564-SHP1 associated with THEMIS after TCR stimulation and SHP1 carrying mutated Tyr536 and Tyr564 behaved functionally similar to SHP1 wt. Thus, our study uncovers a previously unrecognized mechanism by which SHP1 can be recruited to the plasma membrane not directly by its SH2 domains (e.g., via ITIMs), nor via GRB2-SH2 but in complex with a pseudo-adaptor molecule, such as THEMIS. Inspection of the SHP protein sequences did not reveal any obvious and conserved proline-rich sites that can be tested to try and map the GRB2-N-SH3 interaction site in SHP proteins. An SH3-mediated interaction between proline-rich motifs in the C-SH2 and PTPase domains of SHP1 and the adaptor protein CrkL has been described recently (Evren *et al*, 2012). However, we tested by mutational analysis those sites but found no effect on the GRB2 association. Recent 3D structural and mutational studies have revealed an unexpected versatility of SH3 domain binding strategies. These include non-proline-containing sequences that are accommodated by non-canonical binding stratagems within SH3 binding pockets (Saksela & Permi, 2012). Thus, future detailed studies will be needed to map the GRB2 N-SH3 non-canonical site of SHP1 (and likely SHP2). It is remarkable that the spatial arrangement created by GRB2 between SHP and THEMIS (just one of its many partners), where GRB2-SH2 is free to interact with LAT, has not been found thus far in other receptors that use SHP for signal regulation. Such a unique topology may reflect a highly dedicated facet of SHP versatility to achieve subtle signalling regulatory effects in thymocyte selection and T-cell activation.

SHP1 and SHP2 are relatively well expressed in DP and SP thymocytes and mature T cells (BioGPS (http://biogps.org) and Gjorloff-Wingren *et al*, 2000). Earlier studies suggested that SHP1 negatively regulates TCR-proximal signalling by dephosphorylating signalosome components and participates in the control of SP thymocyte development (Zhang *et al*, 1999; Lorenz, 2009). Recently, this conclusion has been challenged by the finding that CD4-cre-mediated SHP1 conditional deletion showed no apparent alteration of thymocyte development, although an increase of memory T cells skewed towards a Th2 phenotype was found (Johnson *et al*, 2013). However, the possibility that SHP2 is redundant in the DP to SP thymocyte transition remained untested. Also, SHP1 gene ablation might have been incomplete. Future investigations of conditional KO of SHP1 and SHP2 alone or combined, at the thymocyte DP developmental stage should help resolve this issue.

How is SHP phosphatase activity switched on in the context of the TCR-dedicated THEMIS-mediated negative feedback? ITIM motifs are not present on LAT, although the possibility exists that a single phosphorylated tyrosine of LAT could provide binding to the N-SH2 of SHP proteins, removing its hindrance on the catalytic site in the phosphatase domain (Lorenz, 2009). Alternatively, phosphorylation of THEMIS tyrosines occurring upon recruitment to LAT (Paster *et al*, 2013) might provide such an unlocking mechanism. Such a hypothesis could not be tested as mutation of those tyrosines locally perturbs THEMIS conformation affecting GRB2 binding to the nearby poly-proline site (Paster *et al*, 2013); hence, recruitment onto LAT and phosphorylation do not occur. However, rather than testing point mutations of SHP1 domains (e.g., SH2 domains) or in THEMIS that may have undesirable side-effects, we aim to test the mechanism of SHP1 catalytic activation in the context of THEMIS association by a direct and simplified approach, by reconstituting the complex *in vitro* using recombinant proteins.

Negative feedback mechanisms in signalling networks reduce output from defined modules/nodes and thus help maintain cellular functions within a “customary” and narrow range (Amit *et al*, 2007). The TCR does not encounter customary ligands but rather, within a set range, a diverse, quasi-continuum, repertoire of affinities and ligand abundance (MHC-bound self- and foreign peptides). Thus, there might be need for the TCR signalosome to modulate signal output to attain stereotypical and highly reproducible gene expression responses. A continuous rectification of variable incoming signals to render them “customary”, may avoid undesired cellular events (e.g. cell death, lineage deviation). During T-cell development, such a device must be promptly inactivated to allow thymocytes to be able to undergo negative selection at high TCR signal strength. Double-negative feedback mechanisms do exist for this purpose (Ferrell, 2002), that alone or coupled to positive feedback offer the possibility for robust digital responses, akin to the narrow choice between life and death of DP thymocyte selection (Gascoigne and Palmer, 2011). Although valid, such a conceptual framework may be an over-simplification, as the *in situ* situation of the thymic microenvironment is more complex and appears to strongly influence the way developing thymocytes perceive incoming ligands of different affinities (Melichar *et al*, 2013).

Earlier work by Germain and co-workers proposed an elegant mechanism by showing that upon pMHC weak agonist engagement, LCK binds via its SH2 domain to phosphorylated SHP1, to dynamically dampen the TCR signalling directly at its inception (Stefanova *et al*, 2003). Moreover, they proposed that Erk activated by pMHC agonists could unlock such a mechanism by phosphorylating LCK on Ser59, which would oppose SHP binding, providing therefore the double-negative feedback device necessary to enact ligand discrimination. The THEMIS:SHP-mediated negative feedback mechanism that we have proposed with *in vivo* demonstration of its role in positive selection (Fu *et al*, 2013) and in the current work in a Jurkat T-cell model and in human T cells may be conceptually similar to that model. However, we notice that our experimental evidence did not detect LCK bound to the THEMIS:GRB2:SHP complex in quantitative THEMIS pull-downs followed by very sensitive immunoblot or MS analyses. Such an interaction should be detected reasonably well if mediated by LCK-SH2 binding to phosphorylated SHP1 as originally proposed (Stefanova *et al*, 2003). We also did not detect an effect of perturbation to the THEMIS:GRB2:SHP complex on LCK activity (pY394) at steady state or after TCR stimulation, but rather an effect on CD3-ζ phosphorylation after TCR stimulation. More importantly, a mouse KI mutation at Ser59 did not demonstrate the effect expected according to the model proposed by Germain and co-workers, both in a developmental setting and in *in vitro* stimulation of T cells carrying a LCK-Ser59Ala mutation, notably not finding the predicted aberration in TCR ligand discrimination. Finally, we did not observe the predicted effect that Erk inhibition should decrease TCR-induced signal propagation (e.g., protect from pMHC-induced apoptosis in the 1G4 system).

The model proposed by Stefanova *et al* implies that SHP1 translocation to the plasma membrane is ensured by active LCK, the only form of LCK—“open”—that can offer the SH2 to bind to phosphorylated SHP1. Recent work has demonstrated that in normal T cells and thymocytes, a sizable proportion (∽40%) of LCK is present in its active form at steady state at the plasma membrane (Nika *et al*, 2010). In this scenario, one should expect SHP1 to bind to active LCK in resting cells. Even if such binding would occur only after TCR stimulation, SHP1 action would not be very effective as most active LCK would be spread over the entire cell surface, and not preferentially where active signalling is happening, as the THEMIS:GRB2:SHP device presumably does. Together, these data suggest that the model proposed by Germain and co-workers is different from the one enacted by the THEMIS:GRB2:SHP1 complex, which has been proven by *in vivo* evidence to be required for establishing the fine threshold between positive and negative selection, hence ligand discrimination.

## Materials and Methods

### Plasmids and antibodies

Full-length cDNA encoding human THEMIS was obtained from Open Biosystems (NM_001010923.2; giving rise to a 641 aa protein: UniProt Q8N1K5-1) and used as the PCR template to generate THEMIS-Strep, carrying a C-terminal One-STrEP-Tag (IBA BioTAGnology). THEMIS-Strep was cloned into the lentiviral expression vector pHR-SIN-BX-IRES-Emerald (kindly provided by Dr. V. Cerundolo, WIMM, Oxford) to give rise to pHR-THEMIS-OST. All mutants described were based on pHR-THEMIS-Strep and derived by site-directed mutagenesis (QuickChange II Kit, Agilent Technologies). THEMIS knock-down/re-expression constructs are based on Tet-pLKO-Puro (Addgene 21915, Dr. Dmitri Wiederschain, Novartis Developmental and Molecular Pathways, Cambridge, MA, USA). Briefly, shRNA-128476 (90% KD efficiency for human THEMIS) was cloned under the control of the H1/TO promoter to give rise to the doxycycline-inducible construct Tet-pLKO-Puro-128476. Exchange of the tetR gene by THEMIS-Strep (wild-type and mutants of the PPR1 site) carrying shTHEMIS-128476 resistance mutations (silent mutations at wobble position of two consecutive codons within the shRNA-target sequence) lead to construct pLKO-Puro-128476-THEMIS-Strep. C-terminal Myc-tagged human GRB2 wt in lentiviral pHR-SIN-BX-IRES-Emerald was described earlier (Paster *et al*, 2013). GRB2 N-SH3 (W36K) and C-SH3 (W193K) mutants were derived by site-directed mutagenesis and cloned in pHR-SIN-BX-IRES-Emerald. Plasmid pcDNA3.1-HA-SHP1, coding for human SHP1 with an N-terminal HA-Tag, was a kind gift of Prof. Ge Baoxue (Institute of Health Sciences, Shanghai Jiao Tong University School of Medicine). All constructs were verified by sequencing. The lentiviral helper plasmids psPAX2 (Addgene 10703) and pMD2.G (Addgene 12259) were provided by Dr. Didier Trono (Ecole Polytechnique Fédérale de Lausanne, Lausanne, Switzerland) via Addgene. Lentiviral shRNA constructs were purchased from Sigma: pLKO-shCtr (SHC002V), pLKO-shSHP1 (TRCN0000244305) and pLKO-shTHEMIS (TRCN0000128476 and TRCN0000130264). Mouse mAbs used included as follows: Alexa647-conjugated anti-phospho-CD247 (Tyr142, clone K25-407.69, BD Biosciences); anti-CD247 (clone 6B10.2, Santa Cruz Biotechnologies); anti-human CD28 (clone CD28.2, Biolegend); anti-human CD3ε (clone UCHT1, eBioscience); APC-conjugated anti-CD69 (clone CH/4, Invitrogen); Alexa647-conjugated anti-phospho-MAPK (ERK1/2, pThr202/pTyr204, clone E10, Cell Signaling Technology); anti-MAPK (ERK1/2, clone 3A7, Cell Signaling Technology); anti-phospho-MAPK (ERK1/2, pThr202/pTyr204, clone 197G2, Cell Signaling Technology); anti-LCK (clone 3A5, Santa Cruz); anti-SHP1 (clone D-11, Santa Cruz); anti-SHP2 (clone M163, Abcam); anti-One-STrEP-Tag mAb (StrepMAB Classic Cat no: 2-1509-001, IBA bioTAGnology; clone GT 661, MyBioscource); anti-phosphotyrosine (clone 4G10, Millipore); anti-Actin (clone AC-15, Sigma); and anti-THEMIS (clone Q13-1103, BD Biosciences). Rabbit polyclonal Abs used were as follows: anti-GRB2 (C-23, Santa Cruz); anti-phospho CD247 (Tyr142, clone EP265(2)Y, Epitomics); anti-HA-Tag (C29F4, Cell Signaling Technology); anti-Myc-Tag (Cat no: 2272, Cell Signaling Technology); anti-SHP1 (clone C-19, Santa Cruz); anti-phospho SHP1 (Tyr564 clone D11G5, Cell Signaling Technologies); anti-phospho-Src-family (pTyr416, Cat no: 2101, Cell Signaling Technologies); anti-GST (Cat no: 2622, Cell Signaling Technology); and anti-THEMIS (Cat no: HPA031425, Sigma). Anti-HA agarose conjugate (mouse monoclonal clone HA-7) was purchased from Sigma.

### Cell lines, transfections and lentiviral transductions

Jurkat 1G4-CD8 cells (expressing the 1G4 TCR and CD8αα) were maintained in RPMI 1640 (PAA Laboratories, Inc.) medium supplemented with 10% foetal bovine serum (FBS, Perbio). J.CaM1.6 LCK-Tet (K. Nika, A. Schulze, T. Hoefer, M. D'abramo, A. Grottesi, L. Schermelleh, O. Acuto, manuscript in preparation), stably carrying a tetracycline-inducible human LCK expression construct, was established using the two-vector Lenti-X Tet-On Advanced inducible Expression System (Clonetech). LCK expression was induced with 1 μg/ml doxycycline for 24 h. Human embryonic kidney epithelial cells (HEK293) were maintained in DMEM, 10% FBS. HEK293 cells were transfected by standard calcium phosphate precipitation. Lentiviral particles were produced in HEK293 cells by co-transfection of lentiviral expression vectors with the packaging plasmids psPAX2 and pMD2.G. 48 h after transfection, viral supernatants were harvested, filtered and used for the transduction of cells in the presence of 5 μg/ml Polybrene. Puromycin selection was applied 48 h post-transduction where appropriate at 1 μg/ml. All parental cell lines are originally from ATCC, tested negative for mycoplasma within previous 3 months and are not STR profiled.

### Preparation of NY-ESO-1 tetramers

Residues 1–278 of the A2 heavy chain with the COOH-terminal BirA tag and β2m were expressed in *E. coli* as inclusion bodies and purified, and refolded to pMHC monomers in the presence of NY-ESO-1 peptides (Cambridge Peptides) listed in Supplementary Fig S2A. Biotinylated monomers were purified by FPLC on an S200 column and tetramerized with phycoerythrin-conjugated streptavidin (Fluka) as described earlier (Altman & Davis, 2003).

### Mass spectrometry data acquisition and analysis

Data were converted to.mzXML format using MSconvert (Proteowizard) and uploaded into the Central Proteomics Facility Pipeline (CPFP) (Trudgian *et al*, 2010) for analysis. Enzyme was set to trypsin allowing for up to 2 missed cleavages. Carbamidomethyl cysteine was set as a fixed modification and oxidation (methionine), deamidation (NQ), acetylation (Protein-N) and phosphotyrosine as variable modifications. Mass tolerances for MS and MS/MS peak identifications were 20 ppm and 0.1 Da, respectively. InterProphet probability (IP Prob) is derived by the combination of results from multiple search engines within CPFP and improves coverage and confidence over use of a single search engine. Label-free quantitation was performed using the SINQ (Spectral index quantitation) tool within CPFP (Trudgian *et al*, 2011). Only proteins which were >20-fold abundant over the biotin-blocked control sample were considered for further analysis using the CRAPOME contaminant repository (Mellacheruvu *et al*, 2013). Finally, proteins found in less than 5% of all Strep-Tag background pull-down experiments listed within CRAPOME were considered as true interactors.

### MS data deposition

The mass spectrometry data from this publication have been submitted to the ProteomeXchange Consortium (Vizcaino *et al*, 2014) via the PRIDE partner repository (http://www.ebi.ac.uk/pride) with the data set identifier PXD001410.

### shRNA-mediated gene knock-down in human CD4^+^ T cells

For each shRNA construct, lentiviral supernatants from three 100-mm dishes of HEK293 cells (10 ml each) were pooled at 48 h post-transfection, filtered through a 0.45-μm syringe filter and subjected to ultracentrifugation at 23,000 × *g* (SW28 rotor) for 90 min at 4°C. Viral pellets were gently resuspended in 300 μl serum-free DMEM medium and either used immediately for transductions or snap-frozen in liquid nitrogen and stored at −80°C for future use. Briefly, human peripheral CD4^+^ T cells were isolated from blood by negative selection using the Dynabeads Untouched Human CD4 T-cell isolation kit (Life Technologies Corp.). 2.5 × 10^7^ CD4^+^ T cells were stimulated per well of a 6-well plate by plate-bound CD3 mAb (UCHT-1, 10 μg/ml), soluble CD28 mAb (CD28.2, 1 μg/ml) and recombinant human IL-2 (50 U/ml; AbDSerotec) for 5 h prior to transduction. Cells were then harvested, suspended in 800 μl RPMI, 10% FBS plus 200 μl concentrated lentiviral supernatant, 5 μg/ml Polybrene, 1 μg/ml CD28 mAb and 50 U/ml IL-2 and reapplied to a CD3 mAb coated 6-well for overnight incubation. Cells were harvested next day and incubated with Human T-activator CD3/CD28 beads (Life Technologies) at a bead:cell ratio of 1:5 in the presence of 50 U/ml IL-2.

### Immunoprecipitations

For SHP1 immunoprecipitation assays, Jurkat 1G4-CD8 cells were lysed in ice-cold lysis buffer (25 mM Tris pH 7.4, 150 mM NaCl, 1 mM EDTA pH 8.0, 1% Triton, 1.75% Octyl β-D-glucopyranoside (Affymetrix), 1 mM Na_3_VO_4_, protease inhibitor cocktail (Roche)). Lysates were cleared by centrifugation at 14,000 × *g* for 10 min. Lysates were pre-cleared with Protein A/G agarose beads (Santa Cruz) for 45 min, and antibody immunoprecipitation was carried out for 2 h using 2 μg of SHP1 (rabbit polyclonal; Santa Cruz C-19) or rabbit IgG antibody, respectively. Protein complexes were pulled down with Protein A/G agarose beads for 2 h, followed by three washes in ice-cold lysis buffer and elution by boiling in SDS sample buffer. For HA IPs, anti-HA agarose (clone 4A6, Millipore) conjugate was used.

### Streptactin pull-down assays and protein interaction analysis by mass spectrometry

Streptactin pull-downs were performed as described recently (Paster *et al*, 2013). Where indicated, protein complexes on Streptactin-Sepharose beads were incubated for 30 min at 37°C with 3 units of alkaline phosphatase (calf intestinal phosphatase, New England Biolabs) in phosphatase buffer [10 mM NaCl, 5 mM Tris pH 7.9, 1 mM MgCl_2_, 0.1 mM DTT and protease inhibitor cocktail (Roche)] or phosphatase buffer only. Following washes, proteins were eluted as described before. For MS analysis, resting or anti-CD3-activated Jurkat cells (1 × 10^8^) expressing THEMIS-Strep were lysed and pulled down as described (Paster *et al*, 2013). Biotin-blocked Streptactin-Sepharose beads were used to assess background. Biotin-eluted proteins were boiled in reducing SDS NuPAGE sample buffer (Invitrogen), followed by alkylation with 55 mM iodoactamide (Sigma). In-gel digest and MS data acquisition were performed as described (Brockmeyer *et al*, 2011).

### Phospho-flow and apoptosis flow cytometry

For Phospho-flow, 5 × 10^5^ 1G4-CD8 cells were stimulated with 200 nM tetramers in RPMI at 37°C. Samples were fixed immediately in fixation buffer (BD Bioscience) at 37°C for 10 min, washed with PBS, 1% BSA, 0.01% azide before permeabilization with 10% saponin on ice for 30 min (Perm/wash buffer 1, BD Bioscience). Antibody staining was for 1 h at RT in 10% saponin buffer. Flow cytometry was performed on a FACSCalibur (Becton Dickinson), and the experiment was analysed using FlowJo (Tree Star, Inc.). Statistical analysis was performed in GraphPad Prism. To detect apoptosis, 2 × 10^5^ 1G4-CD8 cells were stimulated with 20 nm pMHC tetramers or 2 μg/ml camptothecin for 24 h at 37°C in RPMI with 10% FCS. After incubation, cells were stained with FAM- or far-red-FLICA (Immunochemistry Technologies). For plate-bound stimulations, biotinylated 6V monomers were immobilized at the indicated concentrations on Streptavidin-coated 96-well plates (Thermo Scientific) at 4°C overnight in PBS. Plates were washed twice with PBS, and 10^5^ 1G4-CD8 cells/well were incubated ON at 37°C in RPMI with 10% FCS. Cells were harvested, washed in PBS and stained with Annexin-V-Alexa647 (Life Technologies) in 100 mM HEPES, 140 mM NaCl, 25 mM CaCl_2_, pH 7.4. Flow data were analysed as described above.

### Mice

Wild-type, *Lck*^S59A^ (this paper), transgenic OT-I (Hogquist *et al*, 1994) and *Rag2*^−/−^ (Shinkai *et al*, 1992) mice were maintained in specific pathogen-free conditions. OT-I mice expressing wild-type LCK or LCK^S59A^ proteins were maintained on a *Rag2*^−/−^ background. All experiments involving mice were done in accordance with French and European guidelines for animal care.

### Construction of *Lck*^S59A^ knock-in mice

#### Vector construction

A genomic fragment containing all the exons of the *Lck* gene was isolated from a BAC clone of C57BL/6 origin (n° RP23-209C6, Deutsches Ressourcenzentrum für Genomforschung). The TCC codon found in exon 2 of the *Lck* gene and coding for the serine residue present at position 59 of LCK was converted into a GCC codon coding for an alanine. A *lox*P-tACE-CRE-PGK-gb2-*neo*^r^-*lox*P cassette (NEO; Mingueneau *et al*, 2008) was introduced in the intron separating exons 8 and 9 of the *Lck* gene, and a thymidine kinase expression cassette (TK) was abutted to the 5′ end of the targeting vector (Supplementary Fig S6A).

#### Isolation of recombinant ES clones

After electroporation of Bruce 4 C57BL/6 ES cells (Kontgen *et al*, 1993) and selection in G418 and ganciclovir, colonies were screened for homologous recombination by Southern blot. The 5′ single-copy probe corresponded to a 486-bp genomic fragment containing exons 5 and 6 (Supplementary Fig S1A). When tested on *AseI-*digested DNA, it hybridized to a 14.7-kb wild-type fragment or to a 13.7-kb recombinant fragment. The occurrence of an appropriate homologous recombination event at the 3′ side was screened using a 3′ single-copy probe corresponding to a 480-bp fragment of the Neomycin resistance cassette (amplified using primers 5′-GGGAAGGGACTGGCTGCTATTG-3′ and 5′-GCGATACCGTAAAGCACGAGG-3′). When tested on *BglI-*digested DNA, it hybridized to a 10-kb recombinant fragment.

#### Production of mutant mice

Mutant ES cells were injected into FVB blastocysts. Germline transmission led to the self-excision of the NEO selection cassette in male germinal cells. Presence of the intended mutation was checked in homozygous *Lck*^S59A^mice by sequencing a 434-bp genomic fragment encompassing the targeted chromosomal region and using primers 5′-GAATTCAGAGACAAGGTTCAACCA-3′ and 5′-AAGTCTCCATCATGGGAGGGCTCA-3′. Screening of mice for the presence of the *Lck*^S59A^ mutation was performed by PCR using the following oligonucleotides: 5′-CCTCCCCAGGGATTAAAGCTGTGCATC-3′ and 5′-GCCTGTGTCTTCTTCCCAGAGACCTGA-3′. This pair of primers amplifies a 400-bp band in the case of the wild-type allele and a 479-bp band in the case of the *Lck*^S59A^ allele. Mice harbouring the *Lck*^S59A^ mutation (international strain designation C57BL/6-*Lck*^tm3Mal^) are available upon request from EMMA (https://www.infrafrontier.eu) under ID number 04456.

#### Flow cytometry and intracellular staining

The following antibodies were used: anti-CD5 (53–7.3), anti-CD4 (RM4-5), anti-CD8α (53–6.7), anti-TCRβ (H57-597), anti-CD44 (IM7), anti-CD3 (145-2C11), anti-CD24 (M1/69), anti-Vα2 (B20-1) all from BD Biosciences and anti-CD25 (PC61.5) from BioLegend. Cell viability was evaluated using SYTOX Blue (Life Technologies). For intracellular staining of LCK, cells were permeabilized using the BD Cytofix/Cytoperm™ Fixation/Permeabilization Kit according to manufacturer protocol. Anti-LCK antibody (clone 3A5; Santa Cruz) and an isotype control antibody (mouse IgG2b from BD Biosciences) were used at 10 μg/ml. A FITC-conjugated goat anti-mouse IgG2b secondary antibody (Southern Biotechnology) was used to reveal the primary antibody. Stained cells were analysed using an LSRII system (BD Biosciences). Data were analysed with Diva software (BD Biosciences), and overlayed plots were constructed with FlowJo software.

#### OVA peptides and tetramers

OVA peptide variants and a null peptide VSV were synthesized and purified as described (Daniels *et al*, 2006). Biotinylated, soluble peptide-MHC monomer involving the H-2K^b^ molecule and OVA peptide variants were tetramerized with streptavidin as described (Daniels *et al*, 2006).

### Mouse T-cell isolation and stimulation

T cells were purified from pooled lymph nodes and spleens of wild-type and *Lck*^S59A^ mice with Dynabeads Untouched Mouse T cell kits (Life Technologies) with a cell purity of over 90%. Purified cells were stimulated for 72 h with plate-bound anti-CD3 (145-2C11; American Type Culture Collection) with or without soluble anti-CD28 (37–51; American Type Culture Collection). Intracellular IL-2 and IFN-γ were measured as described (Mingueneau *et al*, 2008). Proliferation was measured using CFSE labelling. After 3 days, the absolute numbers of T cells having diluted CFSE were counted after adding Flow-Count™ Fluorospheres (Beckman Coulter).

### OT-I cell stimulation

Splenocytes from *Cd3e*^Δ5/Δ5^ mice (Malissen *et al*, 1995) were used as antigen-presenting cells (APC) and loaded with VSV or the OVA peptide variants described above. Briefly, *Cd3e*^Δ5/Δ5^ splenocytes were irradiated (3,000 rad) and pulsed for 2 h at 37°C with serial dilution of OVA peptide variants. Peptide-loaded APCs were incubated with lymph node T cells from Tg OT-I *Rag2*^−/−^ or *Lck*^S59A^ Tg OT-I *Rag2*^−/−^ mice and tested for CD69 up-regulation and TCR down-modulation. TCR down-modulation was followed by FACS by measuring the decrease of Vα2 expression and was calculated according the formula: % TCR down-regulation = 100 × (*X*/*Y* × 100) where *X* is the mean fluorescence of the TCR expressed when stimulated with APCs pulsed with the OVA or Q4 peptide and Y is the mean fluorescence of the TCR expressed when stimulated with APCs pulsed with a peptide that is not recognized by the OT-I TCR. In the case of stimulation with peptide-MHC tetramers, serial dilution of H-2K^b^-OVA or H-2K^b^-Q4R7 tetramers were incubated with CFSE-loaded lymph node T cells from OT-I *Rag2*^−/−^ or *Lck*^S59A^ OT-I *Rag2*^−/−^ mice. After 3 days, the absolute numbers of CFSE^+^ T cells were counted after adding Flow-Count™ Fluorospheres (Beckman Coulter).

## Supporting Information

Supplementary Figure Legends

Review Process File
