# Supplementary figures and images for: A THEMIS:SHP1 complex promotes T-cell survival
Source: EMBO J. 2014 Dec 22;34(3):393–409. doi: 10.15252/embj.201387725 (PMC4339124; doi:10.15252/embj.201387725)

**A****THEMIS PD**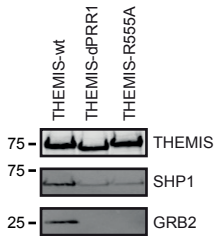**B****Streptactin PD**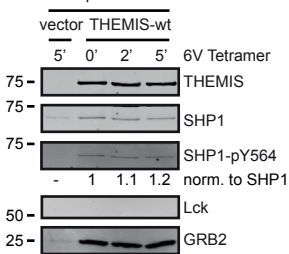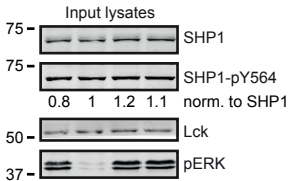**C**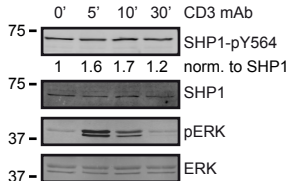**D**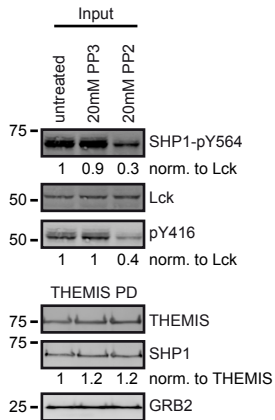

Supplement: Supplementary file 1 [file embj0034-0393-sd1.pdf]

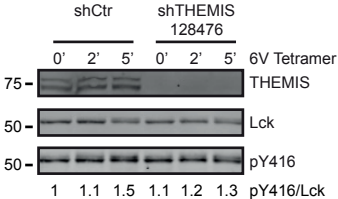

Supplement: Supplementary file 3 [file embj0034-0393-sd3.pdf]

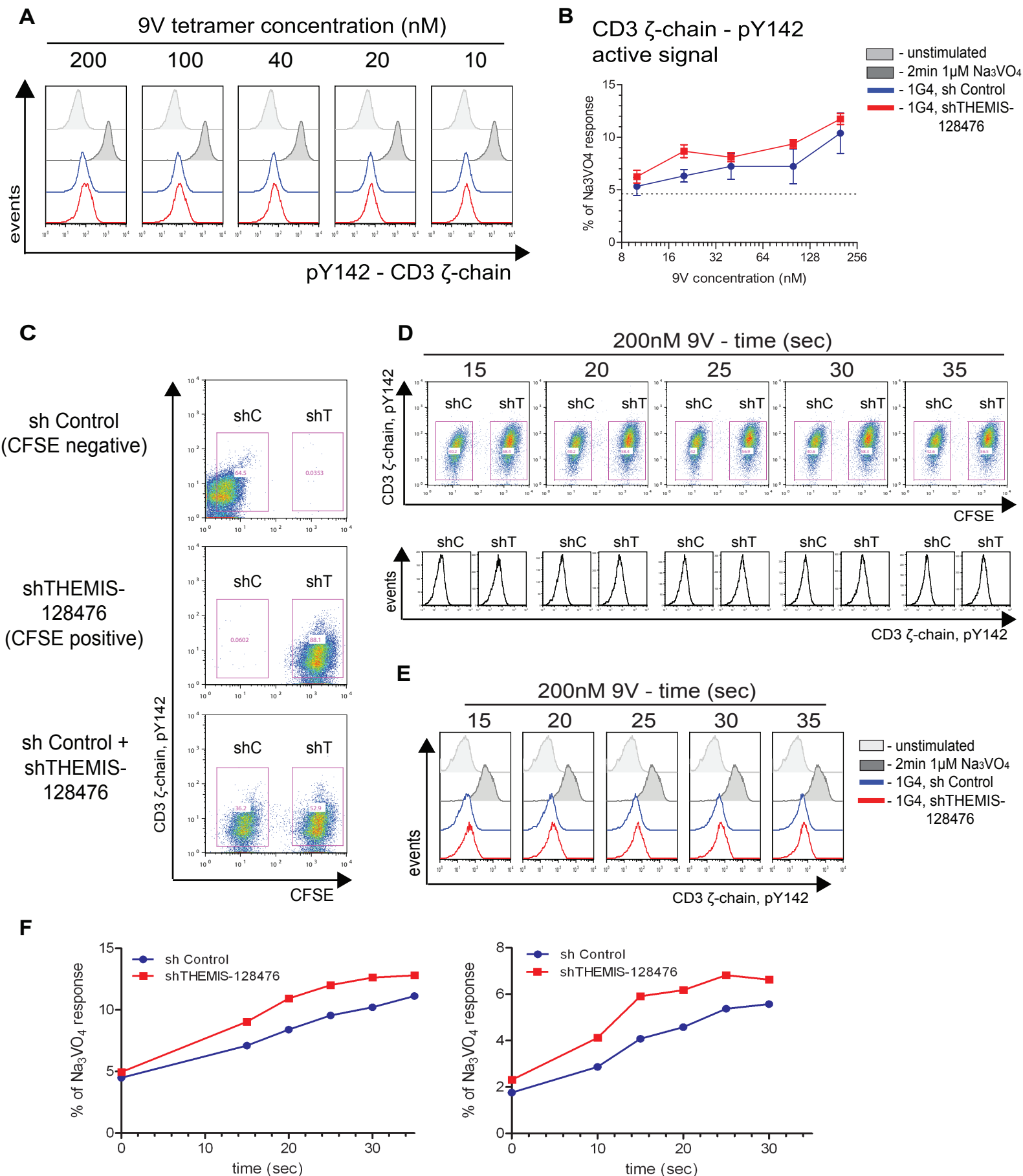

Supplement: Supplementary file 4 [file embj0034-0393-sd4.pdf]

A

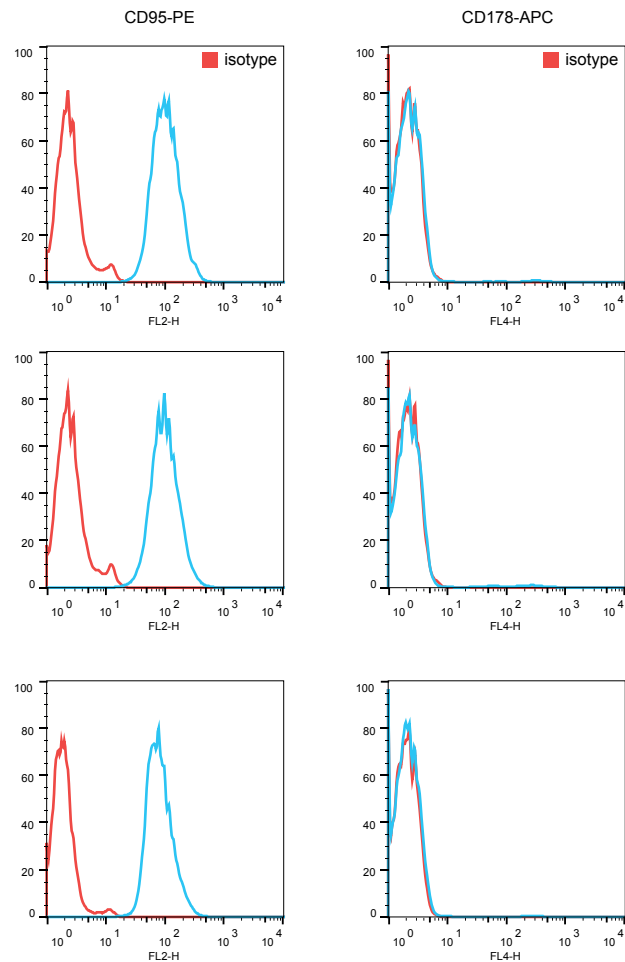

B

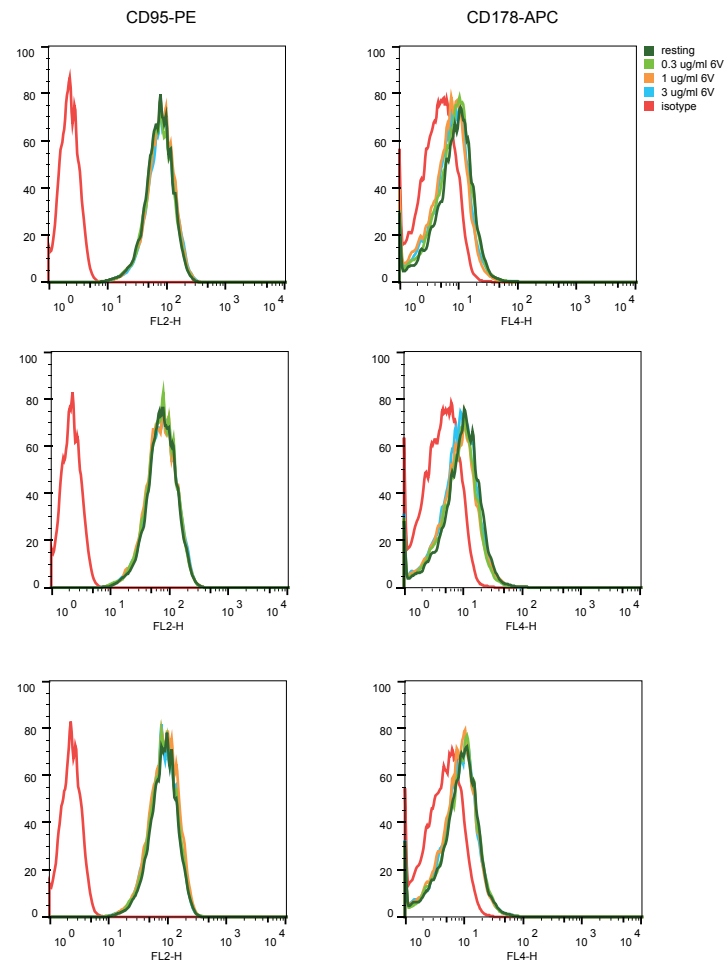

Supplement: Supplementary file 5 [file embj0034-0393-sd5.pdf]

**A**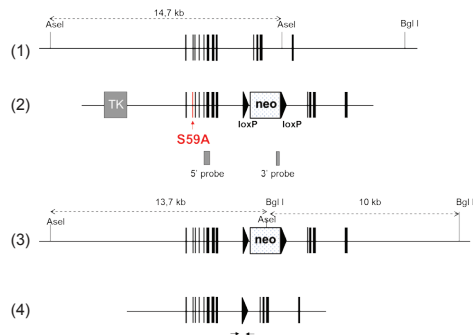**B**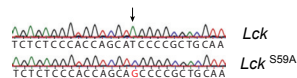**C**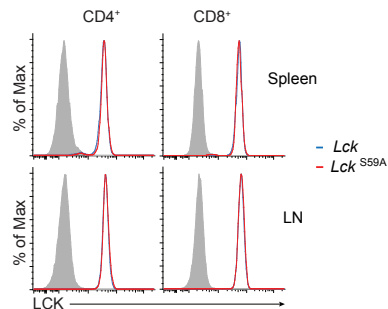**D**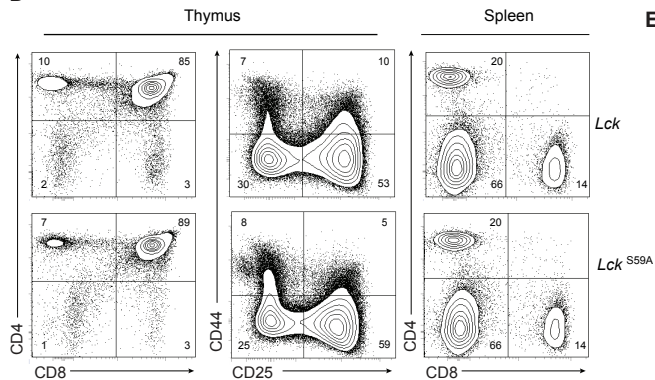**E**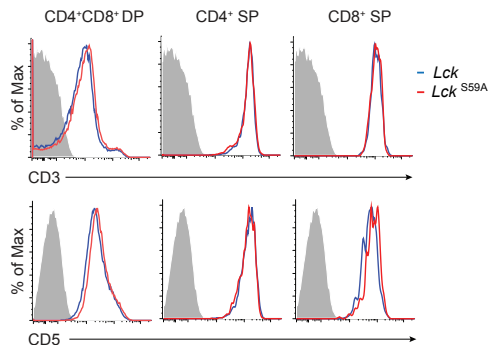

Supplement: Supplementary file 6 [file embj0034-0393-sd6.pdf]

**A**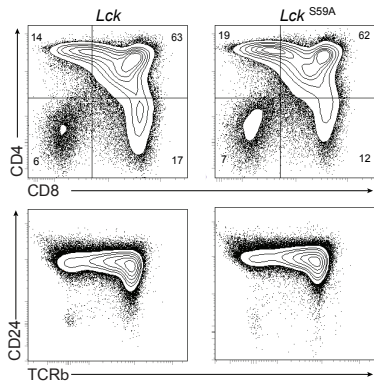**B**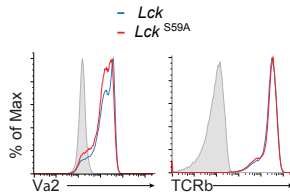**C**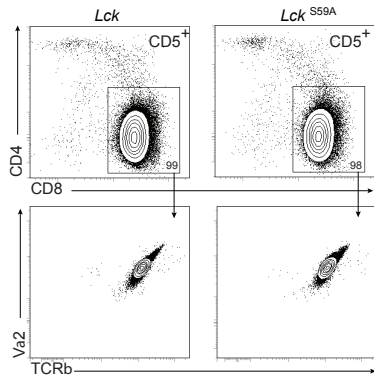

Supplement: Supplementary file 7 [file embj0034-0393-sd7.pdf]

**A**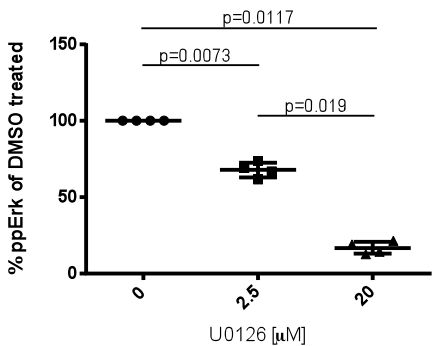**B**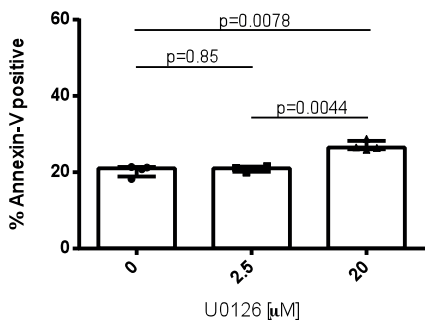**C**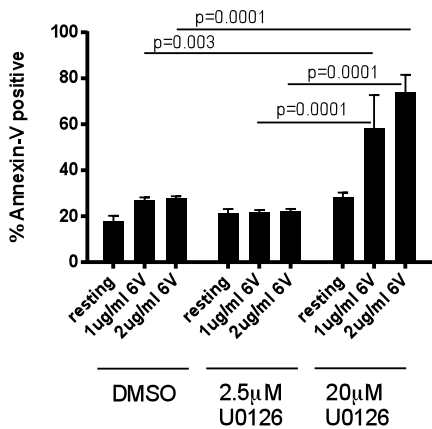

Supplement: Supplementary file 8 [file embj0034-0393-sd8.pdf]
