# Supplementary material for: A THEMIS:SHP1 complex promotes T-cell survival
Source: EMBO J. 2014 Dec 22;34(3):393–409. doi: 10.15252/embj.201387725 (PMC4339124; doi:10.15252/embj.201387725)

A

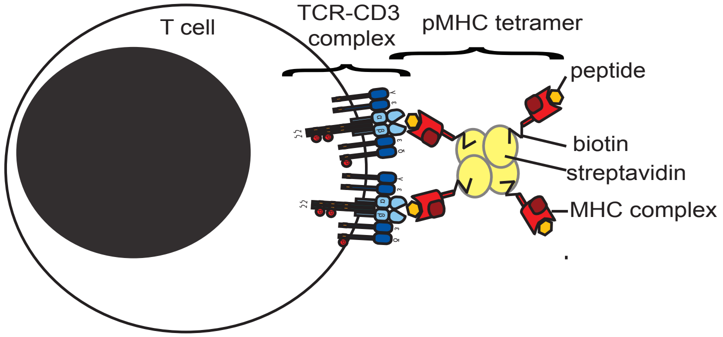

NY-ESO-1$_{157\text{-}165}$ pMHC		K_D (µM)	k_{off} (µM)	k_{on} x10^3 ($M^{-1}s^{-1}$)	$t_{1/2}$ (s)	ΔG (kcal mol^{-1})	EC50 (IFN-γ) (µg/ml pMHC)
peptide name	peptide sequence						
9C (wt)	SLLMWITQC	14 ± 1	0.82 ± 0.01	57 ± 3	0.84 ± 0.01	-6.9 ± 0.0	115 ± 14
9V	SLLMWITQV	7.2 ± 0.5	0.33 ± 0.01	45 ± 4	2.12 ± 0.08	-7.3 ± 0.0	180 ± 19
6V	SLLMWVTQV	18 ± 0	0.85 ± 0.03	49 ± 2	0.81 ± 0.03	-6.8 ± 0.0	45 ± 5
9L	SLLMWITQL	56 ± 6	0.93 ± 0.05	17 ± 2	0.74 ± 0.04	-6.0 ± 0.1	426 ± 113
4D	SLLDWITQV	252 ± 12	2.59 ± 0.15	10 ± 1	0.27 ± 0.02	-5.1 ± 0.0	2.3 ± 0.5

B

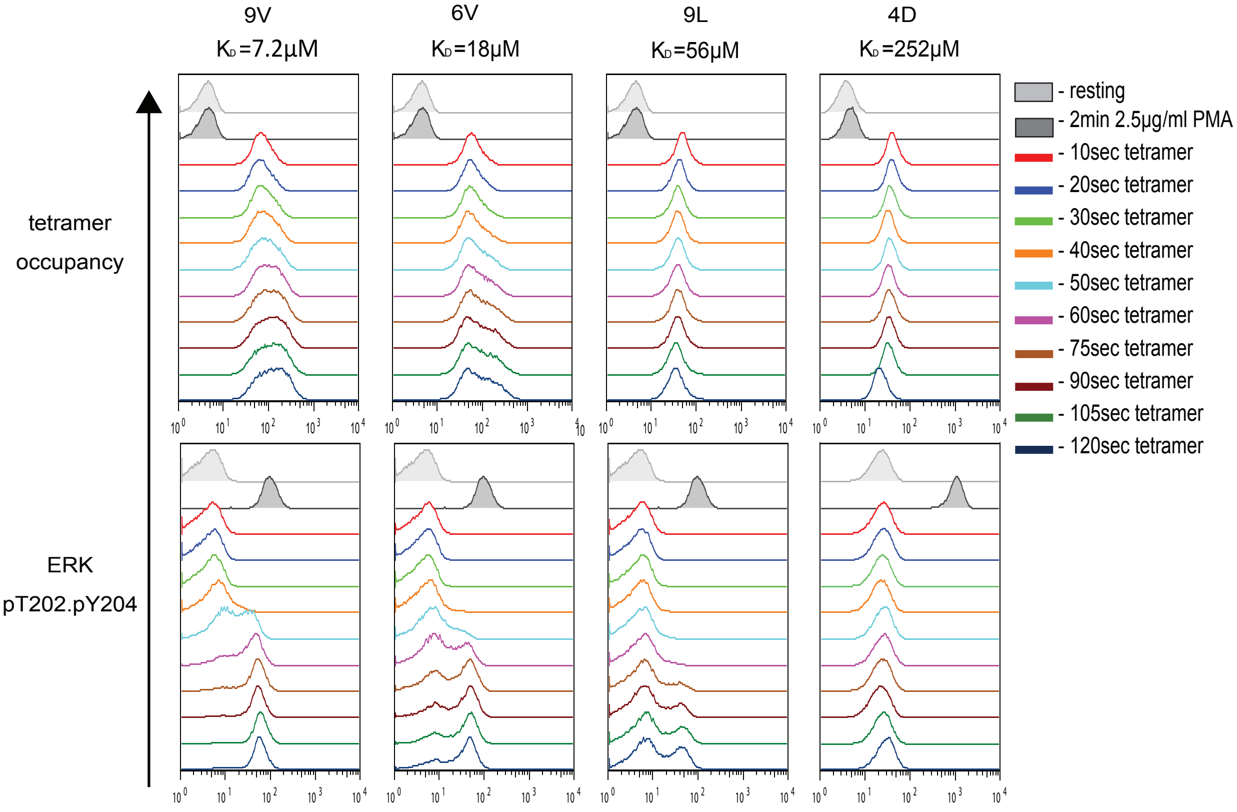

C

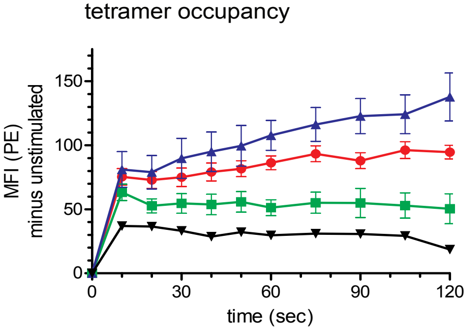

D

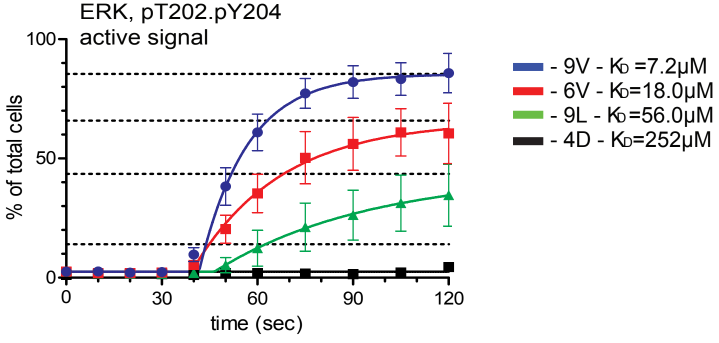

Supplement: Supplementary file 2 [file embj0034-0393-sd2.pdf]
