# Supplementary material for: A THEMIS:SHP1 complex promotes T-cell survival
Source: EMBO J. 2014 Dec 22;34(3):393–409. doi: 10.15252/embj.201387725 (PMC4339124; doi:10.15252/embj.201387725)
Supplement: Supplementary file 9 [file embj0034-0393-sd9.pdf]

Supplementary Table I. Individual peptides indentified in THEMIS PD

THMS1_HUMAN

Position	Probability	Sequence / Charge / PTM	Number of instances
2-15	0.9999	ALSLEEFVHSLDLR	10
88 - 103	0.9999	IVADKTPYLTMEEITR	6
93 - 103	0.9999	TPYLTMEEITR	6
104 - 111	0.9999	TIHIGPSR	8
112 - 121	0.9999	LGHPCFYHQK	8
125 - 131	0.9999	LENLIIK	1
157 - 180	0.9999	NHQTHSFNLPLSQEGEFYECEDER	3
199 - 217	0.9999	TVNLTDFSNKWDSTNPFPK	2
209 - 217	0.9999	WDSTNPFPK	2
218 - 236	0.9999	DFYGTLILKPVYEIQGVMK	5
244 - 253	0.9999	ILPSLDVEVK	6
280 - 305	0.9999	EFPIVTEVIEAPEGNHLPQSILQPGK	5
306 - 312	0.9999	TIVIHKK	5
318 - 324	0.9999	ILASEIR	4
330 - 339	0.9999	RHFLIPTSYK	4
331 - 339	0.9999	HFLIPTSYK	4
348 - 359	0.9999	EFPTAYDLEIAK	3
360 - 371	0.9999	SEKEPLHVVATK	11
372 - 379	0.9999	AFHSPHDK	4
372 - 405	0.9999	AFHSPHDKLSSVSVGDQFLVHQSETTEVLCEGIK	2
380 - 405	0.9999	LSSVSVGDQFLVHQSETTEVLCEGIK	2
407 - 415	0.9999	VVNVLACEK	1
419 - 441	0.9999	KSYEAALLPLYMEGGFVEVIHDK	2
420 - 441	0.9999	SYEAALLPLYMEGGFVEVIHDK	4
420 - 442	0.9999	SYEAALLPLYMEGGFVEVIHDKK	2
443 - 451	0.9999	QYPISELCK	5
509 - 521	0.9999	LNMTVQLVSNFSR	7
522 - 529	0.9999	DAEPFLVR	5
530 - 544	0.9999	TLVEEITEEQYYMMR	8
545 - 559	0.9999	RYESSASHPPPRPPK	9
546 - 559	0.9999	YESSASHPPPRPPK	12
546 - 567	0.9999	YESSASHPPPRPPKHPSVEETK	5
560 - 567	0.9999	HPSVEETK	5
560 - 577	0.9999	HPSVEETKLTLLTLAEER	1
587 - 594	0.9999	RHHVDITK	3
588 - 594	0.9999	HHVDITK	3
588 - 595	0.9997	HHVDITKK	2
595 - 606	0.9999	KLHPNQAGLDSK	17
596 - 606	0.9999	LHPNQAGLDSK	4
607 - 620	0.9999	VLIGSQNDLVDEEK	6
607 - 622	0.9999	VLIGSQNDLVDEEKER	8

GRB2_HUMAN

11-20	0.9999	ATADDELSFK	2
27 - 38	0.9999	VLNEECDQNWYK	2
39 - 50	0.9999	AELNGKDGFIPK	1
51 - 64	0.9999	NYIEMKPHPWFFGK	1
68 - 76	0.9999	AKAEEMLSK	3
101 - 109	0.9999	FGNDVQHFK	1
118 - 124	0.9999	YFLWVVK	2
125 - 136	0.9999	FNSLNELVDYHR	1
196 - 207	0.9999	GACHGQTGMFPR	6

PTN11_HUMAN

6-23	0.9999	WFHPNITGVEAENLLLTR	1
130 - 138	0.985	GKHGSFLVR	1
167 - 173	0.9999	VTHVMIR	1
179 - 186	0.9999	YDVGGGER	1
236 - 244	0.9998	LAETTDKVK	1
281 - 289	0.9994	NILPFDHTR	1
390 - 399	0.9999	ESAAHDYTLR	1

PTN6_HUMAN

10-21	0.9999	DLSGLDAETLLK	2
47 - 55	0.9999	VGDQVTHIR	1
130 - 138	0.9999	GEPWTFLVR	1
295 - 310	0.9677	DSNIPGSDYINANYIK	1
524 - 532	0.9999	KLEVLQSQK	2

COR1A_HUMAN

384 - 393	0.9999	DAGPLLISLK	1
439 - 449	0.9999	KLQATVQELQK	3
454 - 461	0.9688	LEETVQAK	1

M1IP1_HUMAN

12-20	0.896	HSLFNAMNR	1
86 - 95	0.9999	DMYSHYVLLK	1
153 - 160	0.9999	GLHTVLSK	1
