# Supplementary material for: A THEMIS:SHP1 complex promotes T-cell survival
Source: EMBO J. 2014 Dec 22;34(3):393–409. doi: 10.15252/embj.201387725 (PMC4339124; doi:10.15252/embj.201387725)
Supplement: Supplementary file 10 [file embj0034-0393-sd10.docx]

**Supplementary Figure Legends**

**Figure S1. The THEMIS:SHP1 complex is GRB2-dependent.** (**A**) Jurkat 1G4-CD8 cells were transduced with knock-down/re-expression constructs for THEMIS-wt-Strep, THEMIS-dPRR1-Strep and THEMIS-R555A-Strep (specifically targeting the PxRPxK motif in THEMIS). Streptactinpull-downs of THEMIS were analysed for SHP1 and GRB2 by immunoblotting.

(**B**) Jurkat 1G4-CD8 cells were transduced with knock-down/re-expression constructs for THEMIS-wt-Strep or vector control (pLKO-shCtr). Cells were stimulated with 6V monomers for the indicated times, lysed and subjected to Streptactinpull-downs. Isolated protein complexes were analysed by immunoblotting as indicated. ERK phosphorylation on the input lysates is shown as stimulation control.

(**C**) Immunoblots of CD3 mAb stimulated donor-derived human CD4^+^ T cells.

Human CD4^+^ T cells were stimulated with CD3 mAb in solution at 10μg/ml for the indicated time points. Lysates were analysed by immunoblotting for SHP1-pY564 and ERK phosphorylation.

(**D**) Jurkat 1G4-CD8 THEMIS-Strep cells were either left untreated or incubated for 1h with 20mM of the Src-kinase inhibitor PP2 or the negative control PP3. THEMIS-Strep was pulled-down and protein complexes were analysed by immunoblotting. The upper panels show the effect of PP2 on Lck-pY394 and SHP1-pY564 in the input lysates. Isolated protein complexes are shown in the lower panels. Relative amounts of SHP1 normalized to the bait THEMIS-Strep are indicated.

**Figure S2. ERK activation as a function of tetramer affinity in the Jurkat 1G4 system.**(**A**) The 1G4 TCR recognizes the class I restricted NY-ESO-1 melanoma peptide SLLMWITQC presented in the context of HLA-A2. The peptide variants used in this study are derived from the wild type 9C (SLLMWITQC) sequence and are listed in order of decreasing affinity. (**B**) 1G4-CD8 cells were stimulated with 200 nMpMHC tetramer for up to 2 min. Histogram flow cytometry read-out. Data represented are an example of three separate experiments. Tetramer occupancy and pT202.pY204-ERK were recorded simultaneously through the use of distinct fluorochromes. ERK activation displays as two distinct peaks correlating to an unresponsive and an activated fraction of cells. (**C**) Data from three independent experiments were used in the analysis in GraphPad Prism. n=3, means±SEM. (**D**) ERK activation was evaluated by the percentage of cells activated. All graphs show the SEM and are representative of three individual experiments.

**Figure S3. Lck-autophosphorylation in Jurkat 1G4 control and THEMIS KD.**(**A**) Jurkat 1G4-CD8 cells were transduced with lentiviral control or THEMIS-128476 shRNA constructs. Cells were stimulated with 6Vtetramers as indicated, followed by lysis and immunoblotting for THEMIS, total Lck and pY416. pY416 was used as a surrogate to monitor pY394 in Lck. The normalized pY416/Lck ration is shown underneath. Data shown are representative of three independent experiments.

**Figure S4**. **CD3-ζ phosphorylation in 9V-stimulated control and shTHEMISJurkat 1G4-CD8 cells.** (**A**) Jurkat 1G4-CD8 control or THEMIS-128476 cells were stimulated with 9V NY-ESO-1 pMHC tetramers between 10 and 200 nM for 45 sec. The data shown are exemplary for three individual experiments. The cells were tested for pY142-CD3-ζ responses using flow cytometry. (**B**) The phosphorylation of CD3-ζ by 9V tetramers was assessed in relation to the phosphorylation induced by the positive control sodium pervanadate. Data from three independent experiments were used in the analysis in GraphPad Prism. n=3, means± SEM, 2-tailed unpaired Student’s t-test, *P< 0.05, **P< 0.01, and ***P< 0.001. (**C**) Jurkat 1G4-CD8 shTHEMIS-128476 cells were stained with the cell tracker dye CFSE and mixed at a 1:1 ratio with unstained shCtr cells. (**D, E**) 9V NY-ESO-1 pMHC tetramer stimulation was carried out on the pooled cells and the CFSE stain was used to distinguish the two cell populations during analysis. pY142-CD3-ζ stains and analysis was carried out as in (A) and (B). (**F**) Two representative time-courses of pY142-CD3-ζ from the mixed-populations experiments are shown.

**Figure S5. FasR (CD95) and FasL (CD178) expression remain unchanged in THEMIS and SHP1 KD T cells.** (**A**) Donor-derived human CD4+ T cells were transduced with lentiviral constructs coding for shCtr, shTHEMIS-128476 and shSHP1-244305 and analysed for CD95 (FasR) and CD178 (FasL) surface expression on day 4 post transduction. (**B**) Jurkat 1G4-CD8 cells transduced with lentiviral control, THEMIS and SHP1shRNA constructs were either left non-stimulated or incubated for 5h with the indicated concentrations of plate-bound 6V tetramer. Cells were then harvested and surface expression of CD95 (FasR) and CD178 (FasL) were analysed by flow cytometry.

**Figure S6**. **Generation and validation of knock-in mice homozygous for the *Lck*^S59A^ allele.**

(A) Strategy used to produce an *Lck*^S59A^ allele. (1) Partial restriction map of the *Lck*gene. The *Lck*gene contains 12 exons that are shown as filled black boxes. (2) Targeting vector used for the introduction of the S59A mutation in the second exon of the *Lck*gene. The neo selection cassette is bracketed by *lox*P sites (triangles) and directs its own excision as it passes through the male germline. The 5’- and 3’-single copy probes used to verify proper homologous recombination events by Southern blot analysis are shown. TK: thymidine kinase expression cassette. (3) Structure of the targeted *Lck*^S59A^ allele following homologous recombination. (4) Structure of the *Lck*^S59A^ allele following homologous recombination and Cre-mediated self-excision of the neo cassette. Arrows indicate primers used to screen by PCR for the presence of the *Lck*^S59A^ allele presence.

(B) Comparison of the nucleotide sequence encompassing the site of the intended mutation from B6 control mice and homozygous *Lck*^S59A^ mice. The T to G mutation is shown by an arrow.

(C) CD4^+^ and CD8^+^ T cells isolated from the spleen and lymph nodes (LN) of wild-type (*Lck*) and *Lck*^S59A^ mice were analysed for the intracellular expression of the LCK protein. Grey shaded curves correspond to isotype-matched control antibody. Data are representative of three experiments.

(D) Flow cytometry of thymus and spleen. Cells isolated from wild-type (*Lck*) and *Lck*^S59A^ mice were analysed for expression of CD4 versus CD8 and CD44 versus CD25. In the case of the thymus, numbers adjacent to outlined areas of the CD4-CD8 dot plot indicate percent double-positive (DP) cells (top right), CD4^+^ single-positive (SP) cells (top left), CD8^+^ SP cells (bottom right) and double-negative (DN) CD4^–^CD8^–^ cells (bottom left). In the case of the thymus numbers adjacent to outline areas of the CD44-CD25 dot plot correspond to DN1 (top left), DN2 (top right), DN3 (bottom right) and DN4 (bottom left) cells. In the case of the spleen, numbers adjacent to outlines areas of the CD4-CD8 dot plot correspond to CD4^+^ (top left) and CD8^+^ (bottom right) mature T cells. Data are representative of at least three experiments.

(E) Histograms show CD3 and CD5 expression on DP and CD4^+^ and CD8^+^ SP from thymus of wild-type (*Lck*) and *Lck*^S59A^ mice. Grey shaded curves correspond to isotype-matched control antibody. Data are representative of at least three experiments.

**Figure S7. Normal thymic selection in *Lck*^S59A^ mice.**

(A) Thymocytes from OT-I *Rag2*^−/−^ mice expressing wild-type (LCK) or LCK^S59A^ proteins were analysed for expression of CD4 versus CD8 and TCRβ versus CD24. Numbers adjacent to outlined areas of the CD4-CD8 dot plot are as specified in Figure S6A. The co-expression of TCRβ and CD24 permitted to follow the final stages of intrathymic maturation and showed that it proceeded normally in the presence of mutant LCK^S59A^ proteins. Data are representative of at least three experiments.

(B) Histograms show expression of Va2 and TCRβ chain on total thymocytes of OT-I *Rag2*^−/−^ mice expressing wild-type (LCK) or LCK^S59A^ proteins. Grey shaded curves correspond to isotype-matched control antibody. Data are representative of at least three experiments.

(C) Splenocytes from OT-I *Rag2*^−/−^ mice expressing wild-type (LCK) or LCK^S59A^ proteins were analysed for expression of CD4 versus CD8 and Va2 versus TCRβ. Numbers adjacent to the specified gate indicate the percentage of OT-I CD8^+^ T cells. Data are representative of at least three experiments.

**Figure S8. Induction of apoptosis in 1G4-CD8 cells is not blocked by Erk inhibition**.

(**A**)Efficiency of ERK inhibition by the MEK1/2 inhibitor UO126. 2x10^5^1G4-CD8 cells, pre-treated overnight with the MEK1/2 inhibitor UO126 or DMSO as control, were stimulated with 200 nM 6V tetramers and probed for Erk activation by FACS analysis.

(**B**)Impact of long-term ERK inhibition on steady-state surface Annexin-V. 2x10^5^ 1G4-CD8 cells were cultured for 48h in presence of UO126 or DMSO.

(**C**)Impact of ERK inhibition of TCR-induced apoptosis.

6x10^6^1G4-CD8 cells, pre-treated as in (A), were stimulated on 6V-coated plates in medium containing fresh inhibitor. Cells were probed for apoptosis by Annexin-V staining after overnight incubation. Statistics were performed using one-way ANOVA, * indicates significance; ns = not significant.
