# Supplementary material for: A THEMIS:SHP1 complex promotes T-cell survival
Source: EMBO J. 2014 Dec 22;34(3):393–409. doi: 10.15252/embj.201387725 (PMC4339124; doi:10.15252/embj.201387725)
Supplement: Supplementary file 11 [file embj0034-0393-sd11.pdf]

THE
EMBO
JOURNAL

Manuscript EMBO-2013-87725

A THEMIS:SHP complex promotes T cell survival

Wolfgang Paster, Annika M. Bruger, Kristin Katsch, Claude Grégoire, Romain Roncagalli, Guo Fu, Nicholas R. J. Gascoigne, Konstantina Nika, Andre Cohnen, Stephan M. Feller, Philip C. Simister, Kelly C. Molder, Shaun-Paul Cordoba, Omer Dushek, Bernard Malissen and Oreste Acuto

Corresponding authors: Wolfgang Paster, Oxford University; Bernard Malissen, Centre d'Immunologie de Marseille-Luminy; Oreste Acuto, Oxford University

Review timeline:

Submission date:	18 December 2013
Editorial Decision:	07 January 2014
Appeal:	07 January 2014
Editorial Decision:	19 February 2014
Revision received:	23 October 2014
Editorial Decision:	21 November 2014
Accepted:	21 November 2014

Transaction Report:

(Note: With the exception of the correction of typographical or spelling errors that could be a source of ambiguity, letters and reports are not edited. The original formatting of letters and referee reports may not be reflected in this compilation.)

Editor: Alexander Kohlmaier

1st Editorial Decision 07 January 2014

Thank you for submitting your manuscript ("A THEMIS:SHP complex promotes T cell survival") to The EMBO Journal. We have now considered it carefully within our editorial team and unfortunately come to the conclusion that we cannot offer publication in The EMBO Journal.

While we appreciate the technical quality and diverse experimental approaches in this study, we are not convinced that the presented findings are sufficiently conceptually advancing our understanding to warrant publication in a broad title like The EMBO Journal.

We agree that more precisely mapping the function of THEMIS in attenuating TCR signal output and counteracting TCR activation-induced apoptosis is of significance. We note, however, a significant body of previously published work, including your own work, that diminishes the novelty of some findings presented in the present manuscript: THEMIS has already been shown to function in a feedback that modulates TCR signal transmission and THEMIS has been shown to bind GRB2. Increased dell death in THEMIS mutants has also been shown before and THEMIS has been found associated with SHP-1 before in thymocytes. Finally, the interaction of GRB2 with SHP-1 has been shown before and SHP-1 has been earlier involved in negative regulation of TCR signaling.
Irrespective of this, beyond describing similar phenotypes after individual knock-down of THEMIS, GRB2 or SHP-1, we feel that, on a mechanistic level, the functionality of the THEMIS-GRB2-SHP-

© European Molecular Biology Organization

1 complex per se remains to be more decisively demonstrated. Moreover, problems like the molecular details of complex formation (stoichiometry etc.) remain to be worked out. Given the rather advanced state of knowledge, at this point we would look for this sort of new mechanistic insights in an EMBO Journal article.
I am sorry that we cannot be more positive on this occasion, but I wish you every success in your work.

Appeal 07 January 2014

Thanks for your rapid assessment of our manuscript. It seems that an apparent lack of novelty and lack of a more detailed mechanistic study has not convinced the editors.

The arguments for rejecting straightaway our manuscript without sending it to referees reflect, in my opinion, rather rigid criteria for assessing novelty and some lack of knowledge of the scientific matter in question. The comments below dismiss our work as not being sufficiently (actually not all) original. We would have just reproduced the data published in Nature in December 2013 by our two groups. Moreover, according to the comments below, SHP-1 function in TCR signaling would be so obvious and well established that it is of little value to present data addressing this notion

Irrespective of the fact that our data are still very new and definitively help explain THEMIS (and SHP-1) function in T cell development and activation, It seems that the editor(s) have failed to appreciate our central findings demonstrating for the first time that Themis exerts its function also in mature conventional T cells. This cannot be taken for granted, given that Themis expression is considerably decreased in mature T cells. More generally, our data dissipate any doubt that the thymocyte phenotype could be the consequence of TCR signaling re-wiring during thymocyte development in absence of Themis. This often neglected possibility in the interpretation of the actual primary cause of a KO phenotype (especially in complex developmental processes) can only be excluded by showing, whenever possible, the same phenotype by siRNA silencing. Knowledgeable referees ask for such a demonstration, being notorious that there is a general, (often partisan) tendency to assume that ex-vivo data from mice KO models are "sacrosanct". However, experience has taught that all too often this may not the case.

My laboratory discovered the THEMIS:SHP complex in T cells, establishing convincingly its existence through initial detailed mapping studies presented in our manuscript (not in the Nature paper). Importantly, no direct cause-effect relationship between the presence of SHP-1 in the complex and TCR signal attenuation (as for THEMIS) has been demonstrated yet. The increase of TCR-induced activation markers and apoptosis, shown upon SHP-1 siRNA silencing (and mirroring THEMIS deficiency) as reported in our manuscript provides the first and most convincing support for the notion that SHP association with THEMIS and their recruitment onto LAT, is responsible for TCR signal modulation. This is a key piece of data (not trivial) that was missing in our recent Nature paper. Moreover, we show that the mechanism of signal attenuation operates on the TCR complex itself (zeta phosphorylation), again not demonstrated in the Nature paper.

Past literature (of the 80s-90s) tends to attribute to SHP-1 a role in attenuating TCR signals. However, due to SHP-1 expression in essentially all heamotopoietic cell lineages, in vivo SHP-1-deficiency causes a plethora of effects that have not allowed establish, as a matter of fact, that SHP-1 in indeed responsible for attenuating TCR signals and, importantly, no mechanistic basis existed to support this idea. More importantly, thus far there has been no convincing demonstration if and how SHP-1 could be recruited to the immunological synapse. The data presented in our manuscript (not in the Nature paper, or in previous literature) provide compelling evidence for this notion and that THEMIS (via LAT) mediates this.

So, I do not share the opinion that our work does not make 'sufficiently conceptually advancing [for] our understanding to warrant publication in a broad title like The EMBO Journal". I feel rather than a solid work that strengthen and extends work published only one month ago is of interest to a large audience of scientists working on T cell signaling.

Moreover, I would be very grateful if you could clarify what do you mean by "on a mechanistic level, the functionality of the THEMIS-GRB2-SHP-1 complex per se remains to be more decisively demonstrated". If you have precise suggestions about specific points that need to be addressed on the function of the THEMIS-GRB2-SHP-1 complex, we would be more than happy to answer to the above critics and resubmit a revised manuscript with additional experiments in a near future.

Finally, I would like to add a comment. There is no very compelling reason to submit scientific work of "biblical" proportions. In my decades-long experience of reviewing papers this too often results in stretching volume over quality favouring addition of quantity of (expensive) minutia over conceptual substance, the aim being to provide at any cost a "mechanism", though often failing to really do so. You would agree with me that carefully designed and controlled experiments must be welcome over volume, to establish solid scientific facts.

2nd Editorial Decision 19 February 2014

Thank you for submitting your manuscript "A THEMIS:SHP complex promotes T cell survival".

We have received reports from four trusted referees. The referees all expressed some interest in principle, but raised a number of important concerns about the conceptual advance of the present dataset, in particular over two recent papers in JI and Nature from your laboratory, but also to previously published work by others on the role of SHP-1 in negative feedback regulation of proximal TCR signaling as well as on activation-induced cell death. In the light of the modest extension of novelty of the presented findings, I would like to stress that the current dataset would have to be extended considerably to advance the level of molecular mechanistic insight. In light of this, we have to request in this case that all points raised by the four referees would need to be addressed in the course of a major experimental revision. We understand that this may well be beyond the scope of the current project and we would understand if you therefore decide to publish the paper elsewhere.

As the specific points raised by the referees appear generally well-taken and self-explanatory, I will not go through them in detail here, but briefly summarize some key points that, if addressed, would make the paper more compelling.

A. The mechanisms how THEMIS participates in TCR regulation and how SHP-1 is activated in order to inhibit TCR signaling remain to be determined in molecular detail. This should include delineating the roles of SHP1's SH2 domains and their elusive binding moiety during activation, the role for Y564 in SHP-1 for recruitment to signaling complexes, the role of LAT phosphorylation in this respect, the functional targets of SHP-1 phosphatase, and the amount of active SHP-1 in the complex.
B. Related to this, formal proof that the THEMIS:GRB2:SHP-1 complex is functional as a ternary complex should be provided by mutant reconstitution with more selective mutations that specifically abrogate SHP-1 recruitment to the complex. In this respect, also a clear demonstration that Grb2 must dimerize for the functional complex to form remains to be established.
C. The unexplained modest effect of Themis knockdown on ERK signaling, raised by reviewers 4, 1 and 3: measuring ERK signaling in dependence on Themis knockdown levels on single cell level may address this point.

We generally allow three months as standard revision time. In the light of the nature of the specifics of the experiments requested here, we would in principle be open to extend the deadline if necessary subject to considering the literature closer to the deadline.

Importantly, I also want to point out that it is our policy that competing manuscripts published during this three months period would have no negative impact on our final assessment of your revised study. However, we request that you contact the editor as soon as possible upon publication of any related work, to discuss how to proceed. Should you foresee a problem in meeting the three-month deadline, please let us know in advance, so we are can plan with an extended deadline.

Thank you for the opportunity to consider this work, and please do not hesitate to contact me in case you should have any additional question regarding this decision or the reports.

REFEREE REPORTS

Referee #1:

THEMIS is required for the differentiation of immature CD4-CD8 double positive (DP) thymocytes into mature CD4 or CD8 single-positive (SP) thymocytes. Previous work from the Acuto/Gascoigne laboratories (Fu et al. 2013, Paster et al. 2013) demonstrated that (1) THEMIS is constitutively associated with GRB2, (2) soon after TCR stimulation THEMIS associates with LAT, via the adapter protein GRB2, and in turn TEMIS is rapidly tyrosine-phosphorylated, (3) THEMIS interacts constitutively with the protein-tyrosine phosphatase SHP1 and SHP2, (4) upon recruitment to LAT, the THEMIS-associated SHP1/SHP2 phosphatases become tyrosine phosphorylated and dephosphorylate substrates such as LAT, PLCg1 and LCK. Accordingly, THEMIS performs its function through controlling recruitment and activation of the phosphatases SHP1/SHP2, which limit TCR signalling and affect the thresholds for positive and negative selection. The present manuscript attempts to elucidate the basis of the THEMIS-SHP1 association and the functional impact of SHP recruitment. Using a THEMIS bait (THEMIS-Strep) expressed in Jurkat cells, the authors confirmed by MS that THEMIS is constitutively associated to GRB2, SHP1 and SHP2. By expressing a shRNA-resistant mutant of THEMIS-Strep and a shTHEMIS hairpin in Jurkat 1G4-CD8 that express an αβ TCR specific for a class I restricted NY-ESO-1 melanoma peptide, the authors succeeded in effectively replacing endogenous THEMIS. Using this system, they again confirmed that THEMIS is part of a constitutive complex that includes
GRB2 and both SHP1 and SHP2. This complex does not seem to be affected by TCR activation. Using HEK293T cells transfected with HA-tagged SHP1 and Myc-tagged GRB2 wild-type or mutants, they established that the C-SH3 domain of GRB2 likely binds to SHP1. This is a puzzling finding taking into account that GRB2 already associates constitutively via its C-SH3 domain to the PxRPxK-motif in the C-terminus of THEMIS. The authors suggest but do not demonstrate that such paradox may be solved provided that GRB2 exists as a dimer. To solidify their previous model (Fu et al. 2013) that SHP1 is transported near the TCR signaling machinery upon TCR ligation and down
modulates the incoming signal, they stimulated Jurkat 1G4-CD8 with peptide-MHC tetramers of decreasing affinity and monitored Erk activation and CD3zeta phosphorylation by cytometry. Stimulation of THEMIS knockdown cells with a weak agonist showed a significant - but very low - increase in phosphorylated Erk. A more impressive increase was achieved in the case of CD3zeta phosphorylation. Therefore, consistent with the recent results published by the authors in Nature, the recruitment of the THEMIS/SHP complex onto LAT enacts a negative feedback mechanism that curtails the TCR signaling cascade from its inception. Furthermore, THEMIS knockdown in CD4 T cells led to increased expression of both CD69 and CD25 and to a higher rate of cell death. Congruent results were obtained with SHP1 knockdown. Altogether these results increment slightly the model of Themis function proposed by the authors a few months ago by showing that SHP is recruited by the C-SH3 domain of GRB2 and that THEMIS-bound SHP dephosphorylates CD3zeta and in turn partially protects T cells from apoptosis.
Specific issues
1/ Are GRB2, SHP1 and SHP2 the only molecules interacting with THEMIS at resting and activated stages ? If they are not, the authors need to present/discuss the full set of constitutive and TCR-inducible interactors.
2/ Figure 1B and its legend are not self-explanatory and need to be made more clear. The endogeneous (mock) Themis and Themis Strep need to be labelled.
3/ In the Introduction and Discussion, the authors discuss several times the importance of negative feedback and digital TCR signal. They should also quote recent data (Melichar et al Science Signaling 6 : ra92) that shows that « in situ » signals output might be far more complex than those documented ex vivo.

Referee #2:

Using a proteomic's approach, Acuto and colleagues identify in this paper the tyrosine phosphatases SHP-1 and SHP-2 as two proteins that interact with THEMIS and adaptor protein found by the authors and other groups to be important for T cell selection in the thymus. Since these 2 phosphatases are known negative regulators of TCR signaling, the authors investigate the possibility that THEMIS negatively regulates T cell activation via the recruitment of both phosphatases. Using biochemical approaches, the authors identify the previously known THEMIS interactor, Grb2, as the protein responsible for recruiting SHP to THEMIS. Therefore, the interaction of SHP with THEMIS is not direct, but mediated by Grb2. The authors make use of different mutants of the Grb2 SH3 and SH2 domains to identify the domains involved in the binding of SHP. They find that the C-terminal SH3 domain of Grb2 plays a major role in the interaction with SHP, with minor participation of the Grb2 SH2 domain as well. The problem is that the C-terminal SH3 domain of Grb2 is the domain that interacts with THEMIS and therefore, it does not seem "a priori" possible that Grb2 interacts simultaneously with SHP and THEMIS. The authors propose that the ternary THEMIS-Grb2-SHP complex could be possible with the participation of Grb2 dimers. The authors study the possible functional relevance of the ternary complex by knockdown approaches. In my opinion, here is where the manuscript requires major revision.

Major concern:

1)Knockdown of SHP and THEMIS indicates that they both negatively regulate T cell activation but it does not demonstrate that the recruitment of SHP to THEMIS is required for such process. In order to demonstrate that the ternary complex is mediating negative signaling, the authors need to define better the site of interaction of SHP on Grb2 to allow the generation of a point mutant that specifically abrogates SHP binding to Grb2. Such a mutant would allow to use a knockdown-reconstitution approach (similar to this used for Fig. 1C) to demonstrate that abrogation of SHP recruitment to Grb2 (but not of Grb2 binding to THEMIS) results in loss of negative regulation of signaling.

The ambiguity in the characterization of Grb2-SHP interaction may have derived from the C-terminal SH3 domain mutant used by the authors. To abrogate the capacity of the N-terminal SH3 domain to bind its ligands, the authors use a conventional mutant (P49L) that eliminates one of the pockets for proline-residue binding in PxxP sequences. However, the C-terminal SH3 domain mutant (G203R) is not at the ligand-binding site of the SH3 domain (see for instance the structure in PDB code 1I06), but in one of the beta-sheets of the domain. I am afraid that the use of this mutant has led the authors to overestimate the importance of this domain in SHP binding. A more conventional mutation, for instance P206A or W193A, could give different results.

Other concerns:

2)Expression of the activation markers CD69 and CD25 by THEMIS knockdown cells (Fig. 4) is not sufficient to evaluate effects on "full" T cell activation. Cytokine release or, even better, T cell proliferation would be more adequate read-outs.

3)The reference to the paper of Daniels et al 2006 for a digital ERK response is not the most appropriate, perhaps referring to this of Germain and colleagues (PLOS Biol 2005) would do justice.

Referee #3:

In the present manuscript, Acuto and colleagues show that a TCR signaling complex involving Themis, Grb2 and SHP-1 is necessary to dampen signaling and subsequently prevent activation-induced apoptosis of human T cells. Most experiments are performed in vitro with transduced Jurkat and human peripheral T cells and the results described here are very clearly presented and discussed.

General comment

This is an important study that dissects the molecular mechanism by which Themis affects early TCR signaling. The protein Themis was originally described as a Grb2 binding factor involved in early TCR signaling. A number of published papers have emphasized its biological importance in vivo by showing that a genetic deletion of Themis profoundly affects thymic selection. A clear picture of how Themis affects thymic selection has only emerged recently through combined findings by the Gascoigne and Acuto laboratories. In the absence of Themis, early TCR signaling is increased especially with low affinity peptide-MHC complexes (Fu et al, Nature 2013). Here, this manuscript elegantly shows how Themis and Grb2 recruit the inhibitory phosphatase SHP-1 to the TCR signalosome and the authors nicely demonstrate the importance of the SH2 and SH3 domains of Grb2 in stabilizing this complex. Intriguingly the association is constitutive and does not increase after TCR activation. As previously shown by the Gascoigne group, knock-down of Themis leads to increased TCR signaling and apoptosis. The authors here nicely confirm these results using a

completely different system. Overall, the present manuscript does provide some explanation on how the absence of Themis may affect thymic selection in vivo by providing a negative feedback loop to decrease TCR signaling

1. I would have liked to see the amount of SHP-1 associated with Themis and Grb2 that is tyrosine-phosphorylated (Figure 1). It is not clear to me how much active SHP-1 is actually bound to this complex before and after TCR stimulation.
2. In Figure 3, knock-down of Themis affects CD3zeta phosphorylation much more dramatically with the stronger 9V tetramer than with the 6V tetramer. There is here a small discrepancy with the ERK phosphorylation experiments described in Figure 2B-C and Figure 3. Analyzing more intermediate steps during TCR signaling (using Jurkat cells stimulated with different tetramers) like ZAP-70 and/or LAT phosphorylation as well as Calcium fluxes may help resolve this discrepancy.
3. In Figure 4, I would have liked to see a titration curve with human T cells stimulated by anti-CD3 antibodies on day 4 after stimulation. The amount of CD69 upregulation with control cells seems low. A titration would highlight the increased signaling observed with reduced levels of Themis and further strengthen their observation.

Referee #4:

In this submission, the authors extend work contained in two 2013 papers from the same group, one in JI and the other in Nature, already showing that THEMIS forms complexes with Gb2, LAT, and SHP-1 and that the latter contributes to attenuating TCR signals in response to antigen engagement to limit cell death evoked by strong signaling (in the previous work, mainly with respect to thymocytes).

Major issues:

1) In the present paper, the authors claim to uncover a novel mechanism of TCR signal control, but this claim is rather disingenuous as made clear by both the papers cited from Lorenz (and older ones that could have been noted from Matt Thomas and the cited Stefanova et al. work in Nat Immunology). The involvement of SHP-1 in negative feedback regulation of proximal TCR signaling in CD4+ mature T cells is well documented. At best, the new results relate to evidence for THEMIS involvement in that process, but this is really already a major conclusion of the Nature paper, albeit with thymocytes, and by itself is a rather modest advance. Likewise, the complex of THEMIS with Grb2, the role of the SH3 domain in this complex formation, and even the role of LAT are already covered in the JI paper and not new pieces of information.

2) As to the specific experiments presented here, the analysis of the role of phosphorylation and SH2 domains in the recruitment of SHP-1 to the TCR complex during feedback regulation are incomplete. While the inhibitor study shown in Fig. 1D suggest that the THEMSI- GRB2 complex is not Y-P dependent, as the authors admit, the effect of the inhibitor is incomplete, and more to the point, the relevant aspect of the mechanism of SHP-1 mediated inhibition of TCR signaling per se is not revealed by this analysis. SHP-1 and SHP-2 are reported to need SH2 domain interactions to relieve an intramolecular block to the active site of the phosphatase - hence, at some point, these molecules must see a tyrosine phosphorylated substrate to be active in reducing TCR signaling. One mechanism for how this is accomplished is reported in Stefanova et al., though the authors have not considered these data in the design of their experiments. Further, their own data on LAT interactions indicate that phosphorylation of the LAT adapter is involved in the set of interactions involving THEMIS-GRb2- SHP1, but this is not followed up in the present study.

3) The data on ERK activation treat ERK signaling as only a downstream outcome of TCR proximal signaling, but there are also reports of Erk acting back on components of the proximal TCR signaling apparatus as well (see the Altan-Bonnet paper cited by the authors). Erk analysis is thus complicated by this recursive feedback loop function. Beyond the lack of any discussion of this complexity, the actual data in Figure 2 show only a modest effect of THEMIS KD on Erk signaling under very limited conditions, and mostly with respect to the fraction of responding cells. It would have been preferably and more informative if the authors had used existing methods for simultaneously evaluating protein levels and signaling in the same cells (see Feinerman et al.

Science 321:1081, 2008 for a method for doing this) so that one could relate the extent of THEMIS KD to the response on a per cell basis.

4) It is also strange that the final part of the paper, dealing mainly with cell viability and activation induced cell death, includes no citations to the work of Lenardo and colleagues, who first reported the phenomenon and carefully assessed the role of caspases, Fas and TNF ligands, and BH3 domain proteins in this process. In particular, they have shown that elevated signaling, for example, associated with strong agonists vs. weak ones, has a major impact on the extent of cell death, as does the amount of available ligand. Both results show that strength of signal correlates with the extent of induced death. The authors do check for increased expression of Fas, but do not even note why that is worthwhile; they should be more scholarly in how they treat this topic given that they are mainly confirming existing results that elevated TCR signaling contributes to apoptosis of T cells.

Overall, much of the paper reprises recently published work from this group or very modestly extends it to mature T cells, with some further mapping of protein interactions. The key questions, which relate to how THEMIS participates in regulation of TCR signaling, are only superficially addressed, especially in light of past work already showing the central role of SHP-1 in negative feedback regulation (this is not a novel concept, as the authors state). A much better submission would more carefully dissect the possible role of the SHP-1 SH2 domains in activating phosphatase function, the role of LAT phosphorylation in these events, and the target(s) of SHP-1 phosphatase activity, while also critically testing past models for the specific molecular events involved in feedback regulation, such as the role of Y-564 on SHP-1 in recruitment to signaling complexes in the synapse.

1st Revision - authors' response 23 October 2014

General comments

We notice that all four referees had not raised major concerns about the central notion developed in the present work, namely that in T cells THEMIS binds constitutively to SHP proteins and that, upon TCR stimulation, THEMIS shuttles SHP to the plasma membrane via binding to phosphorylated LAT, in the vicinity of the engaged TCR. Such a negative feedback reduces TCR-proximal signalling and more downstream pathways and, in so doing, diminishes the chances of T cells do undergo apoptotic cell death upon TCR stimulation. We have discussed the mechanistic role of Themis and SHP in T cell survival and activation threshold. This mechanism is likely to play a central role in TCR ligand discrimination. Our previous work published in December 2013 in Nature showed that a similar mechanism operates in DP thymocytes but no detail of the underlying biochemical mechanism was developed in that work. We provided evidence that THEMIS is required to guarantee effective positive selection of conventional T cells, since its absence compromises TCR ligand discrimination, which helps setting a fine threshold between positive and negative selection. We now provide a more detailed account of the underlying mechanism of negative feedback enacted by SHP1 via THEMIS to serve the purpose of fine ligand discrimination. Future work, just recently started in our laboratories (N.R.J.G and O.A), will address further the *in vivo* relevance of these findings and proposed mechanisms in the thymus and periphery using THEMIS and SHP conditional gene KO approaches.

The present work reveals that THEMIS-depletion in a T cell model and in normal human T cells results in a mild, yet reproducible and significant augmentation of TCR-induced zeta phosphorylation, Erk activation, as well as later activation events, such as increased CD69 and CD25 expression. A relevant consequence of such a generalised TCR signal increase, likely less well controlled than in THEMIS-sufficient T cells, is that T cells are more prone to activation-induced apoptotic cell death.

Although all four the referees did not raise major concerns about technical issues or flawed experimental design, they all felt that the work would gain in novelty, amplitude and robustness, if we addressed more precisely the biochemical basis for the formation of the THEMIS:SHP complex mediated by GRB2, the role of SHP C-terminal tyrosine and SH2 domains in driving recruitment of SHP onto LAT and SHP activity regulation. Moreover, besides some minor incorrect, misplaced or missed bibliographic citations, referee 4 suggested in addition to compare and contrast mechanistically our proposed THEMIS:GRB2:SHP-mediated

TCR-induced negative feedback to the elegant and conceptually similar model proposed already in 2003 by R. Germain and co-workers to explain TCR ligand discrimination. Such a negative feed back combined with a double-negative feedback mechanism was proposed by Germain and Altan-Bonnet in 2005 to be a requirement for better explaining TCR ligand discrimination, as a tout-court "proof-reading" model proposed by McKeithan in 1995, would be insufficient to do so. Finally, some concerns were raised by two referees about the relatively mild effects of THEMIS depletion on TCR-proximal signalling and the apparent substantial increase in apoptosis.

We have now addressed all the points raised, adding a new set of experiments, discussed why the signalling effects could be mild and the phenotypic effects apparently more accentuated and corrected ambiguous or missing bibliographic citations. We have also addressed with experimental evidence the comparison of the R. Germain and co-workers to the mechanism we describe here.

Point-by-point answer to the referees

Comments referee #1:

THEMIS is required for the differentiation of immature CD4-CD8 double positive (DP) thymocytes into mature CD4 or CD8 single-positive (SP) thymocytes. Previous work from the Acuto/Gascoigne laboratories (Fu et al. 2013, Paster et al. 2013) demonstrated that (1) THEMIS is constitutively associated with GRB2, (2) soon after TCR stimulation THEMIS associates with LAT, via the adapter protein GRB2, and in turn TEMIS is rapidly tyrosine-phosphorylated, (3) THEMIS interacts constitutively with the protein-tyrosine phosphatase SHP1 and SHP2, (4) upon recruitment to LAT, the THEMIS-associated SHP1/SHP2 phosphatases become tyrosine phosphorylated and dephosphorylate substrates such as LAT, PLCg1 and LCK. Accordingly, THEMIS performs its function through controlling recruitment and activation of the phosphatases SHP1/SHP2, which limit TCR signalling and affect the thresholds for positive and negative selection. The present manuscript attempts to elucidate the basis of the THEMIS-SHP1 association and the functional impact of SHP recruitment. Using a THEMIS bait (THEMIS-Strep) expressed in Jurkat cells, the authors confirmed by MS that THEMIS is constitutively associated to GRB2, SHP1 and SHP2. By expressing a shRNA-resistant mutant of THEMIS-Strep and a shTHEMIS hairpin in Jurkat 1G4-CD8 that express an αβ TCR specific for a class I restricted NY-ESO-1 melanoma peptide, the authors succeeded in effectively replacing endogenous THEMIS. Using this system, they again confirmed that THEMIS is part of a constitutive complex that includes GRB2 and both SHP1 and SHP2. This complex does not seem to be affected by TCR activation. Using HEK293T cells transfected with HA-tagged SHP1 and Myc-tagged GRB2 wild-type or mutants, they established that the C-SH3 domain of GRB2 likely binds to SHP1. This is a puzzling finding taking into account that GRB2 already associates constitutively via its C-SH3 domain to the PxRPxK-motif in the C-terminus of THEMIS. The authors suggest but do not demonstrate that such paradox may be solved provided that GRB2 exists as a dimer. To solidify their previous model (Fu et al. 2013) that SHP1 is transported near the TCR signaling machinery upon TCR ligation and downmodulates the incoming signal, they stimulated Jurkat 1G4-CD8 with peptide-MHC tetramers of decreasing affinity and monitored Erk activation and CD3zeta phosphorylation by cytometry. Stimulation of THEMIS knockdown cells with a weak agonist showed a significant - but very low - increase in phosphorylated Erk. A more impressive increase was achieved in the case of CD3zeta phosphorylation. Therefore, consistent with the recent results published by the authors in Nature, the recruitment of the THEMIS/SHP complex onto LAT enacts a negative feedback mechanism that curtails the TCR signaling cascade from its inception. Furthermore, THEMIS knockdown in CD4 T cells led to increased expression of both CD69 and CD25 and to a higher rate of cell death. Congruent results were obtained with SHP1 knockdown. Altogether these results increment slightly the model of Themis function proposed by the authors a few months ago by showing that SHP is recruited by the C-SH3 domain of GRB2 and that THEMIS-bound SHP dephosphorylates CD3zeta and in turn partially protects T cells from apoptosis.

The referee has perfectly summarised our main findings and appreciated our effort to solidify, using *in vitro* systems, the model proposed for Themis function in the thymus with an extension to mature T cells. Because, the mechanistic basis of any *in vivo* phenotype after gene ablation can be ill-interpreted, due to possible compensatory effects (e.g., adaptation by signalling conduits rewiring), *in vitro* systems, if sensibly chosen, can provide substantial and credible mechanistic verification of complex crucial biological phenomena, such as TCR signalling feedback mechanisms. Moreover, they allow to study in much more detail the mechanistic basis of these signalling events to eventually return to *in vivo* experiments to test new hypotheses. We feel that the mechanistic data reported now in the revised version substantially increment the understanding of the

model of THEMIS action, initially drafted in the Fu et al. paper published in Nature in December 2013.

Specific points

1 - "Using HEK293T cells transfected with HA-tagged SHP1 and Myc-tagged GRB2 wild-type or mutants, they established that the C-SH3 domain of GRB2 likely binds to SHP1. This is a puzzling finding taking into account that GRB2 already associates constitutively via its C-SH3 domain to the PxRPxK-motif in the C-terminus of THEMIS. The authors suggest but do not demonstrate that such paradox may be solved provided that GRB2 exists as a dimer.

1 – We have now solved this paradox by re-analysing the formation of the GRB2:SHP interaction using new GRB2 mutants. Referee #2 suggested testing a different mutation at the GRB2 C-SH3, namely W193A, instead of G203R. G203 is not at the ligand-binding site of the SH3 domain but is located in a beta-sheet of the C-SH3 domain. G203R may have long-range/distal structural effects. This very precious suggestion has allowed us to formally eliminate the hypothesis that formation of a GRB2 dimer is needed to explain how THEMIS:SHP complex can associate to pLAT after TCR ligation. Indeed, as shown now in Figure 1F, GRB2 binding to SHP1 was insensitive to the C-SH3 W193A mutation whereas it was sensitive to the N-SH3 W36K mutation, that completely abolished the interaction between GRB2 and SHP1. Previous work (Paster et al. JI 2013) demonstrated that GRB2 C-SH3 binds to THEMIS by recognising a consensus sequence for this class of SH3 domains. For brevity, in the revised version we have omitted to present experiments in 293 cells confirming that GRB2 carrying a W193A mutant does not bind to Themis, confirming, once again, data in Paster et al. JI 2013 that GRB2 C-SH3 is responsible for associating to THEMIS.
Note that, to demonstrate that GRB2 N-SH3 bound to SHP1, we used the W36K mutation, instead of P49L showed in the original version of the manuscript. Inspection of the recent literature (Pawson and co-workers Nature Biotech. (2011) 29:653–658) suggested the former can be more safely utilised than the latter. For brevity, we have omitted to show that a GRB2 double W36K/ W193A mutant also cannot interact with SHP1. Together with the far-western experiments shown in Figure 1G, these data provide now solid evidence that GRB2 binds with the C-SH3 and N-SH3 to THEMIS and SHP1, respectively, leaving the SH2 of the GRB2 molecule in the complex free to interact with LAT, thus removing the need to invoke GRB2 dimer formation. This topology is further substantiated by the demonstration (see Figure 1D, 1E and Figure E1D) that GRB2 SH2 and the C-terminal tyrosines of SHP1 in the particular configuration assumed by GRB2 in the THEMIS:SHP complex do not play any substantial role in the formation of the complex. See pages 7-8 for the new chapters commenting these results: "SHP tyrosine phosphorylation in T cells is dispensable for GRB2-mediated THEMIS:SHP complex formation" and "GRB2 SH3 domains bridge THEMIS to SHP ", and pages 16-17, where we discuss formation of the complex.

2 - Altogether these results increment slightly the model of Themis function proposed by the authors a few months ago by showing that SHP is recruited by the C-SH3 domain of GRB2 and that THEMIS-bound SHP dephosphorylates CD3zeta and in turn partially protects T cells from apoptosis"

2 - In addition to the above data, the revised version contains now the following new data that allow a better understanding of the dynamics, function and regulation of the THEMIS:SHP complex:

2A – We show now that Lck is not part of the THEMIS:GRB2:SHP1 complex before or after TCR stimulation (Figure E1B and Extended data for proteomics analyses). Together with data we provided in the first version of our manuscript that Lck basal phosphorylation is unperturbed in the absence of THEMIS expression (Figure E3 in the revised version), these data reinforces the notion that the TCR-induced THEMIS:GRB2:SHP1-mediated negative feedback acts directly on TCR-p-zeta. Thus, these data make it unlikely that the mechanism we propose is akin to the one suggested by Germain and co-workers in 2003.

2B – We show now that SHP1 C-terminal tyrosines do not play any major role in modulating SHP1 activity. First, we show that phosphorylation of SHP1 C-terminal tyrosines in the pool of SHP1 associated to THEMIS, remain unaltered upon TCR stimulation (see Figure E1B). Second, mutation of both SHP1 C-terminal tyrosines does not affect SHP1 capacity to act as a mediator of the TCR-induced negative feedback (Figure 6E). These two pieces of data also indicate that the THEMIS:GRB2-mediated negative feedback mechanism uncovered by our investigation is distinct from the one proposed by Germain and co-workers in 2003.

2C – In the revised version we show, by a KI mutant mouse model, that Ser59 does not play a significant role in TCR negative feedback (Figures E6, E7 and Figure 7). These data, together with no role found for the

© European Molecular Biology Organization

SHP1 C terminal tyrosine in THEMIS:GRB2:SHP formation and function, indicate that the pathway we report here is entirely novel and distinct from the one previously proposed by Germain and co-workers in 2003.

Because the latter has been thus far the only mechanistic model proposed to help explain TCR ligand discrimination, we feel that the above data presented call for a revision of the way we understand mechanistically this fundamental feature of the TCR signalling machinery. We hope that the new data added to the revised version will convince the referee that now the manuscript does not increment only slightly the model of THEMIS function proposed by the authors in the Nature paper published at the end of 2013.

3 - *Specific issues 1/ Are GRB2, SHP1 and SHP2 the only molecules interacting with THEMIS at resting and activated stages? If they are not, the authors need to present/discuss the full set of constitutive and TCR-inducible interactors are.*

As a matter of fact, it was this initial MS-based evidence gathered in Oxford more than three years ago that led to the idea of a negative feedback mechanism fitting at that time with parallel THEMIS KO and KD observations in Gascoigne's and our laboratory. In the first version of the manuscript, we reported the actual data of that key finding that were not reported in our Nature paper.

3A - As requested by the referee, we have carefully inspected previous MS data and carried out new MS analyses of THEMIS-pull-downs using more rigorously controlled conditions (i.e., THEMIS poly-proline site mutant unable to bind GRB2). Besides background noise, we found no evidence for additional THEMIS specific interaction partners, other than GRB2, SHP1 and SHP2. This is true at steady state and after TCR stimulation. Thus, for instance, we did not detect in subsequent experiments Coronin1A and M1IP1 shown in Table 1 and Table E1. We have however, left these two proteins in the list of Tables 1 and E1 to indicate a cut-off (sequence coverage and spectral counts) below which MS-detection becomes not reproducible, hence untrustworthy. Moreover, we have now commented on page 14 that in the same MS-based experiments we did not find evidence of Lck association to the THEMIS:GRB2:SHP complex. This interaction predicted in the Germain/Stefanova model of signal discrimination, should have been detectable if Lck SH2 interacted with SHP1 C-terminal tyrosines upon TCR stimulation (SH2-mediated interactions are generally of good affinity). This association is also excluded by the immunoblot data of quantitative THEMIS:GRB2:SHP pulldowns data shown now in Figure E1B.

Although we have fully proved that LAT is a partner of the THEMIS-GRB2:SHP complex upon TCR stimulation (Brockmeyer et al., JBC 2011 and Paster et al, JI 2013), detecting LAT by MS is severely limited, if not impossible due to rare Arg and Lys in this protein. In the light of this consideration, studies are underway in our laboratory to inspect THEMIS pulldowns by SILAC-based MS analyses and multiple proteases, to search for potential additional binding partners that are present at steady state and at various time points after TCR stimulation. This work however requires considerable time and it is clearly outside of the scope of the present work.

4 - Stimulation of THEMIS knockdown cells with a weak agonist showed a significant - but very low - increase in phosphorylated Erk

4 – It is not surprising that small changes in signal input may lead to non-linear responses more downstream.

5 - Figure 1B and its legend are not self-explanatory and need to be made more clear. The endogeneous (mock) Themis and Themis Strep need to be labelled.

5 - This has been now corrected in the revised version.

6 - In the Introduction and Discussion, the authors discuss several times the importance of negative feedback and digital TCR signal. They should also quote recent data (Melichar et al Science Signaling 6 : ra92) that shows that « in situ » signals output might be far more complex than those documented ex vivo.

We fully agree with the referee comments that *in vitro* studies are reductionist and cannot reproduce the complexity of *in situ*, cues-rich, environments such as the thymus and peripheral lymphoid organs. We have now cited in an appropriate paragraph of the Discussion (see page 19) the reference suggested.

Comments Referee #2:

We would like to thank dearly the referee for making very constructive critics, helpful suggestions and helping us to solve the puzzle (and an artefact!) of a possible GRB2 dimerisation to explain GRB2 double role in bridging THEMIS to SHP and in the recruitment of the complex onto LAT.

Using a proteomic's approach, Acuto and colleagues identify in this paper the tyrosine phosphatases SHP1 and SHP-2 as two proteins that interact with THEMIS and adaptor protein found by the authors and other groups to be important for T cell selection in the thymus. Since these 2 phosphatases are known negative regulators of TCR signaling, the authors investigate the possibility that THEMIS negatively regulates T cell activation via the recruitment of both phosphatases. Using biochemical approaches, the authors identify the previously known THEMIS interactor, Grb2, as the protein responsible for recruiting SHP to THEMIS. Therefore, the interaction of SHP with THEMIS is not direct, but mediated by Grb2. The authors make use of different mutants of the Grb2 SH3 and SH2 domains to identify the domains involved in the binding of SHP. They find that the C-terminal SH3 domain of Grb2 plays a major role in the interaction with SHP, with minor participation of the Grb2 SH2 domain as well. The problem is that the C-terminal SH3 domain of Grb2 is the domain that interacts with THEMIS and therefore, it does not seem "a priori" possible that Grb2 interacts simultaneously with SHP and THEMIS. The authors propose that the ternary THEMIS-Grb2-SHP complex could be possible with the participation of Grb2 dimers. The authors study the possible functional relevance of the ternary complex could be possible with the participation of Grb2 dimers. The authors study the possible functional relevance of the ternary complex by knockdown approaches. In my opinion, here is where the manuscript requires major revision.

Major concerns:

1 - Knockdown of SHP and THEMIS indicates that they both negatively regulate T cell activation but it does not demonstrate that the recruitment of SHP to THEMIS is required for such process. In order to demonstrate that the ternary complex is mediating negative signaling, the authors need to define better the site of interaction of SHP on Grb2 to allow the generation of a point mutant that specifically abrogates SHP binding to Grb2. Such a mutant would allow to use a knockdown-reconstitution approach (similar to this used for Fig. 1C) to demonstrate that abrogation of SHP recruitment to Grb2 (but not of Grb2 binding to THEMIS) results in loss of negative regulation of signaling.

1 - We agree with the referee that direct evidence is better than an assumption. However, mapping the sequence in SHP proteins responsible for GRB2 binding is not straightforward. The evidence presented in the revised version, thanks to an excellent suggestion by the referee to test W193A, instead of G203R mutation of the C-SH3, has shed new light on the actual topology of the THEMIS:GRB2:SHP complex. It is the N-SH3, and not the C-SH3, that is responsible to anchor SHP to THEMIS (see below). Moreover, new evidence presented in the revised version formally excludes that GRB2-SH2 is involved on the formation of the complex (Figure 1D and E).

A potential SH3-mediated interaction between proline-containing motifs of SHP1 and the adaptor protein CrkL has been apparently mapped (Evren et al, 2012, cited in the manscript). However, we tested by mutational analysis all those three sites for SHP1 binding in 293 cells but did not find an effect on GRB2 association.

In order to locate potential binding sites of GRB2 N-SH3 in SHP1 and SHP2, we had their sequences inspected by Dr. Feller (one of our co-authors in the revised version) and Dr. Kalle Saksela (University of Helsinki), both world-recognised experts in the study on SH3 domain structure and binding specificity. However, they both agreed that SHP proteins do not contain conventional SH3 consensus binding sites and pointed out that there are more and more non-obvious/unconventional SH3 binding sites, lacking typical proline-rich sequences or lacking proline tout-cout, as revealed by recent biochemical and 3D lidand:SH3 crystallographic structures (see cited article: Saksela K, Permi P (2012) SH3 domain ligand binding: What's the consensus and where's the specificity? *FEBS Lett* 586(17): 2609-2614.). It will take a very long investigation to carry out peptide-scanning of the entire SHP sequence to pin down potential sites, carry out mutational analysis, and, to avoid confounding effects, carry out a parallel investigation to prove that the chosen mutations interfere with N-SH3 binding only but do not perturb SHP catalytic regulation. As we have discussed in the revised version, we prefer in future work to understand the formation of the complex and the regulation of SHP activtiy by an in vitro reconstitution assay, as we have now a recombinant form of full-length SHP, GRB2 and THEMIS.

However, we feel now comfortable with the THEMIS:GRB2:SHP topology deduced from our new data that leaves the GRB2 SH2 free to interact with LAT. We consider (the other three referees have not raised this concern) that it would be very unlikely that SHP proteins is recruited onto LAT and then at the moment when they are adjoined to the plasma membrane they are lost (we have not observed significant changes in the amounts of SHP directly associated to THEMIS after TCR stimulation), or their activity was muted. If, the latter possibility was correct, then it will remain unexplained why SHP1 KD phenocopies the functional effects of THEMIS KD. The possibility that other PTPs are implicated cannot be completely ruled out, but the SHP1 KD again makes this possibility less likely.

Rather, the mapping of the GRB2 binding site on SHP would be interesting for future investigations to ask whether the GRB2-induced proximity to THEMIS will reveal a possible influence of THEMIS association on SHP activity, either basally, or after Lck-mediated phosphorylation by of THEMIS. Thus, clearly future in vitro reconstitution study will address this question.

2 - The ambiguity in the characterization of Grb2-SHP interaction may have derived from the C-terminal SH3 domain mutant used by the authors. To abrogate the capacity of the N-terminal SH3 domain to bind its ligands, the authors use a conventional mutant (P49L) that eliminates one of the pockets for proline-residue binding in PxxP sequences. However, the C-terminal SH3 domain mutant (G203R) is not at the ligand-binding site of the SH3 domain (see for instance the structure in PDB code 1I06), but in one of the beta-sheets of the domain. I am afraid that the use of this mutant has led the authors to overestimate the importance of this domain in SHP binding. A more conventional mutation, for instance P206A or W193A, could give different results.

We are particularly grateful for the suggestion by the referee to try other, less disruptive mutations of GRB2 N-SH3, such as W193A, instead of G203R. This very precious suggestion has allowed us to formally eliminate the hypothesis that formation of a GRB2 dimer is needed to explain how THEMIS:SHP1 complex can associate to pLAT after TCR ligation. Indeed, as shown now in Figure 1F, GRB2 binding to SHP1 was insensitive to the C-SH3 W193A mutation whereas it was sensitive to the N-SH3 W36K mutation, that completely abolished the interaction between GRB2 and SHP1. Previous work (Paster et al. JI 2013) demonstrated that GRB2 C-SH3 binds to THEMIS by recognising a consensus sequence for this class of SH3 domains. For brevity, in the revised version we have omitted to present experiments in 293 cells confirming that GRB2 carrying a W193A mutant does not bind to Themis, confirming, once again, data in Paster et al. JI 2013 that GRB2 C-SH3 is responsible for associating to THEMIS.

Note that, to demonstrate that GRB2 N-SH3 bound to SHP1, we used the W36K mutation, instead of P49L showed in the original version of the manuscript. Inspection of the recent literature (Pawson and co-workers Nature Biotech. (2011) 29:653–658) suggested the former can be more safely utilised than the latter. For brevity, we have omitted to show that a GRB2 double W36K/ W193A mutant also cannot interact with SHP1. Together with the far-western experiments shown in Figure 1G, these data provide now solid evidence that GRB2 binds with the C-SH3 and N-SH3 to THEMIS and SHP1, respectively, leaving the SH2 of the GRB2 molecule in the complex, free to interact with LAT, thus removing the need to invoke GRB2 dimer formation. This topology is further substantiated by the demonstration (see Figure 1D, 1E and Figure E1D) that GRB2 SH2 and the C-terminal tyrosines of SHP1 in the particular configuration assumed by GRB2 in the THEMIS:SHP complex do not play any substantial role in the formation of the complex. See pages 7-8 for the new chapters commenting these results: “SHP tyrosine phosphorylation in T cells is dispensable for GRB2-mediated THEMIS:SHP complex formation” and “GRB2 SH3 domains bridge THEMIS to SHP “, and pages 16-17, where we discuss formation of the complex.

3 - Expression of the activation markers CD69 and CD25 by THEMIS knockdown cells (Fig. 4) is not sufficient to evaluate effects on "full" T cell activation. Cytokine release or, even better, T cell proliferation would be more adequate read-outs.

This is a sensible suggestion. However, we would like to argue that if CD69 and CD25 are augmented, why the IL2 production should not? Our data in the present work and in the Nature publication of 2013, indicate that absence of THEMIS results in a generalised augmentation of TCR-directed signalling. When we started this study, we considered testing IL2 production, but only normal T cells as in 1G4 it is hard to detect due to the lack of CD28 expression on those cells. However, having realised that stimulation of 1G4 cells and normal T cells induces a quite substantial cell death relatively early after stimulation, we thought that production of IL2 and cell death will clash, producing then confounding effects difficult to interpret. We have therefore not measured IL2 production in THEMIS KD CD4 T cells, hoping that the referee will share our

view.

4 - The reference to the paper of Daniels et al 2006 for a digital ERK response is not the most appropriate, perhaps referring to this of Germain and colleagues (PLOS Biol 2005) would do justice.

We have now made such a change (see page 9).

Referee #3:

We thank the referee for her/his insights and thought-provoking suggestions. We were very pleased that the referee recognised that "This is an important study that dissects the molecular mechanism by which Themis affects early TCR signalling". And that "… this manuscript elegantly shows how Themis and Grb2 recruit the inhibitory phosphatase SHP1 to the TCR signalosome ….". And that.. "The authors here nicely confirm these results using a completely different system. Overall, the present manuscript does provide some explanation on how the absence of Themis may affect thymic selection in vivo by providing a negative feedback loop to decrease TCR signalling "

In the present manuscript, Acuto and colleagues show that a TCR signaling complex involving Themis, Grb2 and SHP1 is necessary to dampen signaling and subsequently prevent activation-induced apoptosis of human T cells. Most experiments are performed in vitro with transduced Jurkat and human peripheral T cells and the results described here are very clearly presented and discussed.

The referee has perfectly summarised our main findings and appreciated our effort to solidify, using *in vitro* systems, the model proposed for Themis function in the thymus with an extension to mature T cells. Because, the mechanistic basis of any *in vivo* phenotype after gene ablation can be ill-interpreted, due to possible compensatory effects (e.g., adaptation by signalling conduits rewiring), *in vitro* systems, if sensibly chosen, can provide substantial and credible mechanistic verification of complex crucial biological phenomenon, such as TCR signalling feedback mechanisms. Moreover, they allow study in much more detail the mechanistic basis of these signalling events to eventually return to *in vivo* experiments to test new hypotheses. We feel that the data reported in the revised version substantially increment the understanding of the model of THEMIS action, initially drafted in the Fu et al. paper published in Nature in December 2013.

General comment

This is an important study that dissects the molecular mechanism by which Themis affects early TCR signaling. The protein Themis was originally described as a Grb2 binding factor involved in early TCR signaling. A number of published papers have emphasized its biological importance in vivo by showing that a genetic deletion of Themis profoundly affects thymic selection. A clear picture of how Themis affects thymic selection has only emerged recently through combined findings by the Gascoigne and Acuto laboratories. In the absence of Themis, early TCR signaling is increased especially with low affinity peptide-MHC complexes (Fu et al, Nature 2013). Here, this manuscript elegantly shows how Themis and Grb2 recruit the inhibitory phosphatase SHP1 to the TCR signalosome and the authors nicely demonstrate the importance of the SH2 and SH3 domains of Grb2 in stabilizing this complex. Intriguingly the association is constitutive and does not increase after TCR activation. As previously shown by the Gascoigne group, knock-down of Themis leads to increased TCR signaling and apoptosis. The authors here nicely confirm these results using a completely different system. Overall, the present manuscript does provide some explanation on how the absence of Themis may affect thymic selection in vivo by providing a negative feedback loop to decrease TCR signaling .

1 -I would have liked to see the amount of SHP1 associated with Themis and Grb2 that is tyrosine-phosphorylated (Figure 1). It is not clear to me how much active SHP1 is actually bound to this complex before and after TCR stimulation.

1 - We have estimated the amounts of pY-SHP1 associated with THEMIS using accurate far-red fluorescence bands scanning. We divided the intensity pY-SHP1 found associated with > 90% of the THEMIS pool (thanks to a fast and almost quantitative THEMIS pulldown allowed by streptag) by the intensity of the total pY-SHP1 input (e.g., intensity of pY-SHP immunoblot in a fraction of the lysate x by the total volume of the total

lysate). We found that at steady state ~1% of pY-SHP1 was associated with the total pool of THEMIS. The amounts of pY-SHP1 and SHP1 associated to THEMIS remained the same after TCR stimulation (see Figures 1C, E1B). Furthermore, our new data definitively exclude that phosphorylation of SHP1 C-terminal tyrosine play any role on complex formation (see Figure 1D, 1E and Figure E1D). Thanks to an excellent suggestion by referee 2, we have now correctly mapped the association of GRB2 N-SH3 and C-SH3 to SHP and THEMIS, respectively, excluding any role of GRB2 SH2. Moreover, the evidence that mutation of both SHP1 C-terminal tyrosine does not affect SHP capacity to exert a negative role on TCR signalling (Figure 6E), makes very unlikely that C-terminal SHP1 tyrosine phosphorylation plays any significant role in SHP activity modulation in the context of the THEMIS:GRB2:SHP complex.

2 – Furthermore, we would like to clarify the issue of SHP1 and SHP1 phoshorylation as the referee implies that tyrosine phosphorylation necessarily means activated SHP proteins. We would like to offer an alternative view that takes into account our own data in the previous and present version of the manuscript. Although widely assumed, it has never been rigorously demonstrated that the SHP C-terminal tyrosine phosphorylation serves the function of SHP1 (or SHP2) catalytic activation. Or, at least this is unlikely to be the case for the THEMIS-GBR2:SHP complex. The two C-terminal tyrosine-phosphorylation sites of SHP1 and SHP2 are typical SH2-GRB2 consensus binding sites and are likely to play an important role in SHP protein subcellular localisation (via GRB2 SH3-mediated interactions) in signal modulation of certain receptors. However, interestingly this is not the case for the THEMIS:GRB2:SHP complex. GRB2 association to SHP1/2 mediated by its GRB2 SH2 is likely to occur in T cells, as we detect pY-SHP protein at steady state, but this pool may be used for signalling pathways other than the TCR. We have now discussed the unique mode of interactions between members of the THEMIS:GRB2:SHP complex, which has the merit of being reported in this work for the first time and speculate that such a special mode of interactions between the three partners may be an ad hoc adaptation to serve the particular function of SHP to be recruited onto LAT, exactly where active signalling is occurring (e.g., where the TCR is signalling). Previous models proposing recruitment of SHP1 onto Lck via Lck-SH2 domain binding to pY-SHP1 (Germain and co-workers 2003) have the disadvantage of a lesser spatial-selective membrane localisation since active Lck (open conformation free to bind via SH2 domain) is already spread across the entire plasma membrane at steady state as we have previously demonstrated (Nika et al. 2010, Immunity). This is now discussed in the revised version on page19.

3. In Figure 3, knock-down of Themis affects CD3zeta phosphorylation much more dramatically with the stronger 9V tetramer than with the 6V tetramer. There is here a small discrepancy with the ERK phosphorylation experiments described in Figure 2B-C and Figure….

Currently we do not have an explanation for the apparent discrepancies between the effect of THEMIS KD on pErk and p-zeta when testing 9V and 6V peptides. We know that these are assays extremely delicate and it may be difficult to obtain higher accuracy of the measures, the differences being quite small, yet very significant. Qualitatively the pErk and p-zeta data did not contradict each other. Future studies using more sensitive assays may clarify whether the observed differences are due to the phospho-flow analysis itself or reveal subtle and interesting behaviour of different layers of the signalling cascade when removing the SHP negative affect.

4 - *Analyzing more intermediate steps during TCR signaling (using Jurkat cells stimulated with different tetramers) like ZAP-70 and/or LAT phosphorylation as well as Calcium fluxes may help resolve this discrepancy.*

This is in theory an excellent suggestion. Indeed, we did use of pZAP70 and pLAT in phospho-flow applied to THEMIS KD and control cells. They both showed a trend very similar to pERK and p-zeta, namely an increase in both signals in THEMIS KD vs. control cells. However, in our hands both gave high background noise that discouraged us to pursue those experiment for quantitative purposes. We also feel that the additional experiments proposed would not add much more to the central message and novelty of our work.

5 - *In Figure 4, I would have liked to see a titration curve with human T cells stimulated by anti-CD3 antibodies on day 4 after stimulation. The amount of CD69 upregulation with control cells seems low. A titration would highlight the increased signaling observed with reduced levels of Themis and further strengthen their observation.*

We thank the referee for this suggestion. We have now added a titration of anti-CD3/CD28 beads on human T cells deprived of Themis shown in Figure 4D.

Referee #4:

In this submission, the authors extend work contained in two 2013 papers from the same group, one in JI and the other in Nature, already showing that THEMIS forms complexes with Gb2, LAT, and SHP1 and that the latter contributes to attenuating TCR signals in response to antigen engagement to limit cell death evoked by strong signaling (in the previous work, mainly with respect to thymocytes).

Major issues:

1 - *In the present paper, the authors claim to uncover a novel mechanism of TCR signal control, but this claim is rather disingenuous as made clear by both the papers cited from Lorenz (and older ones that could have been noted from Matt Thomas and the cited Stefanova et al. work in Nat Immunology). The involvement of SHP1 in negative feedback regulation of proximal TCR signaling in CD4+ mature T cells is well documented. At best, the new results relate to evidence for THEMIS involvement in that process, but this is really already a major conclusion of the Nature paper, albeit with thymocytes, and by itself is a rather modest advance.*

We have appreciated the authentic intent of the referees to encourage us to try and answer questions concerning the molecular mechanism underlying THEMIS:GRB2:SHP complex formation, the role of SHP1 determinants in regulating its activity and to compare our model to the one proposed by Germain and co-workers in 2003 and 2005.

However, we disagree with some of her/his general comments and some statements. We do not feel of having been disingenuous (a significant assertion implying bad faith) in claiming that the mechanism we describe here is novel. *Sensu stricto*, our claim is correct. It is based on a novel molecular mechanism implicating SHP recruitment via THEMIS:GRB2 onto LAT, a mechanism that acts on TCR zeta rather than on Lck, as our data (old and new) clearly suggest. A mechanism that utilises phospho-LAT and should therefore act more directly and effectively at the membrane region where active TCR signalling is occurring.

In our initial submission, we have scrupulously recognised the earlier contributions by Ulrike Lorenz'and Ben Neel's work, which also made earlier suggestions that SHP1 is a negative regulator of TCR signalling. We omitted citing the earlier work by Matthew Thomas because we felt that the in vivo/ex-vivo studies *motheaten* phenotype and the transgenic PTP-dead overexpression were all cited in the Lorenz's comprehensive review that we cited). We feel that we had the same attitude with Ron Germain's work. Both 2003 and 2005 seminal papers of this group were largely cited. However, we have now more clearly emphasized the merit of Germain and co-workers in proposing the elegant notion implicating SHP1-mediated negative feedback TCR ligand discrimination. Such a mechanism activated only by weak TCR ligands was suggested to act directly on Lck, while an Erk-mediated phosphorylation of Lck Serine59 would offset such a negative effect when the TCR engaged with agonists. Incidentally, we have already recognised in the Nature paper of 2013, the conceptual merit of the Germain's model.

2 – "Likewise, the complex of THEMIS with Grb2, the role of the SH3 domain in this complex formation, and even the role of LAT are already covered in the JI paper and not new pieces of information."

It is unclear here what the referee really meant. An attentive reading of the data reported in the first version of our manuscript would make immediately clear that we have not re-addressed the role of GRB2 SH3 in THEMIS binding, nor of THEMIS binding onto LAT. This was addressed, as the referee recognises, in a previous JI paper, in which we have correctly defined the molecular details of GRB2:THEMIS complex formation and mapped where THEMIS:GRB2 complex binds on phosphorylated LAT and formally demonstrated *in vivo* that the THEMIS interaction with GRB2 is required for its recruitment to the IS and for T cell development. It is only in the present work that we have addressed the precise role of GRB2 in bridging THEMIS to SHP proteins, as this direct link was not at all mentioned in the Nature paper.

In conclusion, we have not claimed (and do not claim now) to having re-discovered that SHP1 contributes to a TCR-induced negative feedback and its importance in ligand discrimination. Rather, we have attempted here to define the molecular details of this novel mechanism. The series of evidences we present now clearly show that the mechanism for which we provide a plausible molecular basis is novel with respect to previously proposed mechanisms.

3 - As to the specific experiments presented here, the analysis of the role of phosphorylation and SH2 domains in the recruitment of SHP- 1 to the TCR complex during feedback regulation are incomplete. While the

inhibitor study shown in Fig. 1D suggest that the THEMSI- GRB2 complex is not Y-P dependent, as the authors admit, the effect of the inhibitor is incomplete,

3 - We have now fully addressed the molecular basis for THEMIS:GRB2:SHP complex formation and define that C-terminal phosphorylation of SHP1 does not contribute to the complex formation. See Figure 1D, 1E and Figure E1D and a new chapter "SHP tyrosine phosphorylation in T cells is dispensable for GRB2-mediated THEMIS:SHP complex formation" and "GRB2 SH3 domains bridge THEMIS to SHP " has now been added to the revised version of the manuscript.

4 - and more to the point, the relevant aspect of the mechanism of SHP1 mediated inhibition of TCR signaling per se is not revealed by this analysis. SHP1 and SHP-2 are reported to need SH2 domain interactions to relieve an intramolecular block to the active site of the phosphatase - hence, at some point, these molecules must see a tyrosine phosphorylated substrate to be active in reducing TCR signaling. One mechanism for how this is accomplished is reported in Stefanova et al., though the authors have not considered these data in the design of their experiments.

4 – Before illustrating the new data that attempted to address the mechanistic basis of SHP inhibition (see below), we would like to raise a few issues. One of the most relevant and novel aspect of the mechanism we propose resides in the fact that THEMIS shuttles SHP proteins onto LAT, thus in very close proximity of the fraction of signalling TCRs. The model proposed by Stefanova et al. suggested that antagonist/weak TCR ligands induce SHP1 translocation to the plasma membrane where it bound to active Lck, the only form of Lck - "open" - that can offer the SH2 to bind to phosphorylated SHP1. Although, not known at the time when Stefanova et al. proposed their model, it is now been demonstrated that in normal T cells and thymocytes a sizable proportion (~ 40%) of Lck is present in its active form at steady state at the plasma membrane (Nika et al. Immunity, 2010). In this scenario, one should expect SHP1 to bind to active Lck at steady state (e.g., without TCR stimulation). Moreover, in such a model, even if SHP1 association with active Lck would occur only after TCR stimulation, SHP1 action may not be very effective as SHP1 would be spread over the entire cell surface and not preferentially where active signalling is happening. We have now added a short comment at the end of the discussion to highlight the potential advantages of the THEMIS-GRB2:SHP-enacted vs. the Lck:SHP1-enacted mechanism.

We agree with the referee that at some point SHP molecules should see a tyrosine phosphorylated substrate to be activated and reduce TCR signalling. The following answers address the SHP1 activation issues raised by the referee and the comparison with the Stefanova-Germain's model.

1 - Using either very sensitive MS analysis or immunoblot, we have found no evidence that Lck is bound to THEMIS:GRB2:SHP1 before or after TCR stimulation (see Figure E1B and MS data).

2 – The German and co-workers model implied that SHP1 would use its two phosphorylated C - terminal tyrosines to interact with Lck-SH2 domain. In so doing, it would act directly on Lck and dephosphorylate tyrosine 394, the Lck activation determinant. However, we have observed no appreciable change in pY394 by immunoblot (Figure E3) (and by FACS analysis, not shown) in cells KD for THEMIS before and after TCR stimulation. Incidentally, the two phosphorylation sites (Y536/Y564) at the C-terminal tail of SHP1 (and SHP2) are excellent consensus sequences for GRB2-SH2 but not for Lck-SH2.

3 – We now show that SHP1 carrying mutated Y536/Y564 (Y536F/Y564F) is functionally indistinguishable from SHP1 wt, when examining TCR-stimulated apoptosis (Figure 6E). These data, together with evidence presented in Figures 1D, 1E and E1D of the revised manuscript indicating that SHP1 C-terminal tyrosines do not play an appreciable role in complex formation make unlikely that these tyrosines play a role in TCR-induced negative feedback mechanism.

4 - We attempted to study the role the two SH2 domains of SHP1 using the same complementation assay of the experiment of Figure 6E. We generated single and double mutants of SHP1 SH2 domains. However, in contrast to the SHP1 Y536F/Y564F mutant, expression of one of the single mutants and the double mutant was substantially weaker than the wt in 1G4 cells, thus precluding any meaningful experiment to address this important issue. Such a decreased expression was observed also in 293 cells. We think therefore that mutating one of the two SH2 domains destabilises SHP1. Consequently, we have taken the view that, rather than studying SHP1 regulation by an indirect and uncontrollable approach, such as site-directed mutagenesis, we will address this issue by in vitro reconstitution experiments and monitoring SHP activity, using recombinant THEMIS, GRB2, SHP1, Tyr-phosphorylated LAT peptides and Lck (to induce phosphorylation of THEMIS).

Together, our data reveal that the THEMIS:GRB2:SHP-mediated TCR-induced negative feedback that is key for signal discrimination and limits T cell activation is built on a mechanism distinct from the one proposed by Germain and co-workers.

5 - Further, their own data on LAT interactions indicate that phosphorylation of the LAT adapter is involved in the set of interactions involving THEMIS-GRb2- SHP1, but this is not followed up in the present study.

5 – In our previous work (Paster et al. JI 2013), we have only demonstrated that THEMIS binds to LAT GRB2-SH2 sites (thus, presumably via the GRB2-SH2 associated to THEMIS). It is incorrect to say that "*their own data*" implicated *"that the LAT adapter is involved in the set of interactions involving THEMIS-GRb2-SHP1"*. Which set of interactions? Indeed, in our previous work we only analysed THEMIS-GRB2 complex as SHP1 was not studied and not even mentioned in that paper. We have never found or characterised interactions of SHP1 with LAT. Instead, our current working hypothesis is that upon recruitment onto LAT via THEMIS, SHP1 could be activated, by its SH2 interacting with tyrosine phosphorylated THEMIS. However, as we explain in the discussion such a hypothesis cannot be tested by mutating THEMIS tyrosine that become phosphorylated upon LAT recruitment as such mutation abolish GRB2 binding to the nearby GRB2 C-SH3 binding site (as shown in Paster et al. 2013). Alternatively, the mode of activation of SHP proteins when bound to THEMIS is unconventional and will require removal of PTP catalytic hindrance by the N-SH2, by a novel mechanism. We have now discussed possible models of SHP protein activation in the context of THEMIS:GRB2:SHP:LAT complex on page 18 and indicated that such studies would be better performed by in vitro reconstitution of the THEMIS:GRB2:SHP:LAT complex.

6 - The data on ERK activation treat ERK signaling as only a downstream outcome of TCR proximal signaling, but there are also reports of Erk acting back on components of the proximal TCR signaling apparatus as well (see the Altan-Bonnet paper cited by the authors). Erk analysis is thus complicated by this recursive feedback loop function. Beyond the lack of any discussion of this complexity, the actual data in Figure 2 show only a modest effect of THEMIS KD on Erk signaling under very limited conditions, and mostly with respect to the fraction of responding cells. It would have been preferably and more informative if the authors had used existing methods for simultaneously evaluating protein levels and signaling in the same cells (see Feinerman et al. Science 321:1081, 2008 for a method for doing this) so that one could relate the extent of THEMIS KD to the response on a per cell basis.

6 – We agree that *"only a modest effect of THEMIS KD on Erk signalling"* is observed. It is important to remind here that it is not surprising that relatively small, but measurable receptor-proximal events, can non-linearly generate downstream substantial biological effects (cell death of a sizable cell population).

Concerning the role of Erk in activating a double-negative feedback by phosphorylating Lck and opposing Lck binding to SHP1, we now provide a series of new data that address this mechanism. We generated KI mice that carry mutated Ser59 (Ser59Ala) and observed no effect on thymus development and only a minor augmentation of TCR signalling with weak pMHC ligands in T cells from Tg TCR OT1 KI mice (Figure 7). If the mechanism proposed by Germain and co-workers was correct, one would have observed an alteration of thymocyte selection as ligand discrimination should be altered. Likewise, TCR signalling upon weak ligation should result in decreased responses. Moreover, we tested whether inhibition of Erk activation in our 1G4 system would reduce apoptotic cell death (as expected if inhibition by SHP1 would be unopposed by the action of Erk). However, depending in the level of Erk inhibition, we found either no effect or increased apoptosis.

We conclude that our data do not support a similarity between the THEMIS:GRB2:SHP1:pLAT-mediated mechanism and prompt a revision of the model by Germain and co-workers. We have now added an additional chapter reporting these data: Mutation of Ser59 of Lck does not affect TCR signalling and ligand discrimination *in vivo and in vitro" and* in the discussion we have added comments on these data and the implications of the results observed (pages 19-20).

7 - It is also strange that the final part of the paper, dealing mainly with cell viability and activation induced cell death, includes no citations to the work of Lenardo and colleagues, who first reported the phenomenon and carefully assessed the role of caspases, Fas and TNF ligands, and BH3 domain proteins in this process. In particular, they have shown that elevated signaling, for example, associated with strong agonists vs. weak ones, has a major impact on the extent of cell death, as does the amount of available ligand. Both results show that strength of signal correlates with the extent of induced death. The authors do check for increased expression of Fas, but do not even note why that is worthwhile; they should be more scholarly in how they

treat this topic given that they are mainly confirming existing results that elevated TCR signaling contributes to apoptosis of T cells.

7 – We have now cited a relevant reference of Lenardo and co-workers that make justice of our initial involuntary omission.

9 - Overall, much of the paper reprises recently published work from this group or very modestly extends it to mature T cells, with some further mapping of protein interactions. The key questions, which relate to how THEMIS participates in regulation of TCR signaling, are only superficially addressed, especially in light of past work already showing the central role of SHP1 in negative feedback regulation (this is not a novel concept, as the authors state). A much better submission would more carefully dissect the possible role of the SHP1 SH2 domains in activating phosphatase function, the role of LAT phosphorylation in these events, and the target(s) of SHP1 phosphatase activity, while also critically testing past models for the specific molecular events involved in feedback regulation, such as the role of Y-564 on SHP1 in recruitment to signaling complexes in the synapse.

8 – We would like to finally stress that our new data do not support the idea that Lck is a target of SHP1 phosphatase activity, but rather it acts directly on TCR-zeta phosphorylation. We think these data address the point raised by the referee on the target reached by SHP1. Moreover, our data make very unlikely that Y536/Y564 on SHP1 are involved in the recruitment to signalling complexes to the IS. Finally, testing past models of TCR-negative feedback required for ligand discrimination indicate that the THEMIS:GRB2:SHP:LAT-mediated mechanism does not fit into previously proposed models. We hope that the referee will recognise that we have made considerable efforts that improve the submission of this work.

3rd Editorial Decision 21 November 2014

Thank you for submitting your revised manuscript "A THEMIS:SHP complex promotes T cell survival" to The EMBO Journal. We have been able to contact three of your four referees, and your manuscript has now been assessed again by the referees. You will find their comments pasted below. I was very pleased to see the modifications introduced and the advances made, and the referees now fully endorse publication of your work. Thus, I am glad to inform you that the EMBO Journal is now accepting your manuscript for publication.

REFEREE COMMENTS

Referee #2:

The authors have resolved my major concerns by demonstrating how Grb2 simultaneously can bind to THEMIS and SHIP, making it possible to bring this negative regulatory complex to the LAT signalosome. The authors have reinforced their data on TCR-proximal phosphorylation events and conclude that the SHP1-Grb2-THEMIS complex is a negative regulator of TCR signaling with effect on thymic selection (diminishes negative selection) and mature T cell activation (inhibits T cell activation including activation-induced cell death.
By generating and reporting the phenotype of a new knockin mouse with a mutation in the Serine residue in position 59 of Lck, the authors conclude that their negative regulator complex is different to the negative feed-back loop proposed by Germain and colleagues, thus introducing a higher degree of complexity to the regulatory loops that fine-tune the TCR signal.
In my opinion the article is ready to be shown to the readers of EMBO J since it shows with elegant proteomics, biochemical and genetic means the composition and effects of a new signal regulatory loop.

Referee #3:

Acuto and co-authors have answered all my concerns and provided significant additional data to support their hypothesis.
Therefore, I recommend publication of this manuscript.
Best regards.

Referee #4:

The authors have done a remarkable amount of additional work to validate and extend the results included in their original submission. They have also directly tested the related model of Stefanova et al. and found that the previously proposed model both differs mechanistically from the present findings in which THEMIS-SHP1 interaction plays an important role in TCR ligand discrimination and also that an in vivo test of the relevant Lck mutation that is central to the prior model does not show strong effects. These new data strengthen the authors' claim that their mechanism is distinct from the one previously reported and also of physiological relevance. The revised paper should be published and available to the field.

© European Molecular Biology Organization
